# Rational Approach to Finding Genes Encoding Molecular Biomarkers: Focus on Breast Cancer

**DOI:** 10.3390/genes13091538

**Published:** 2022-08-26

**Authors:** Nathalie Schneider, Ellen Reed, Faddy Kamel, Enrico Ferrari, Mikhail Soloviev

**Affiliations:** 1 Department of Biological Sciences, Royal Holloway University of London, Egham, Surrey TW20 0EX, UK; 2 School of Life Sciences, University of Lincoln, Lincoln LN6 7TS, UK

**Keywords:** transcription factors, biological pathways, gene expression, microarrays, transcriptomics, molecular biomarkers

## Abstract

Early detection of cancer facilitates treatment and improves patient survival. We hypothesized that molecular biomarkers of cancer could be rationally predicted based on even partial knowledge of transcriptional regulation, functional pathways and gene co-expression networks. To test our data mining approach, we focused on breast cancer, as one of the best-studied models of this disease. We were particularly interested to check whether such a ‘guilt by association’ approach would lead to pan-cancer markers generally known in the field or whether molecular subtype-specific ‘seed’ markers will yield subtype-specific extended sets of breast cancer markers. The key challenge of this investigation was to utilize a small number of well-characterized, largely intracellular, breast cancer-related proteins to uncover similarly regulated and functionally related genes and proteins with the view to predicting a much-expanded range of disease markers, especially that of extracellular molecular markers, potentially suitable for the early non-invasive detection of the disease. We selected 23 previously characterized proteins specific to three major molecular subtypes of breast cancer and analyzed their established transcription factor networks, their known metabolic and functional pathways and the existing experimentally derived protein co-expression data. Having started with largely intracellular and transmembrane marker ‘seeds’ we predicted the existence of as many as 150 novel biomarker genes to be associated with the selected three major molecular sub-types of breast cancer all coding for extracellularly targeted or secreted proteins and therefore being potentially most suitable for molecular diagnosis of the disease. Of the 150 such predicted protein markers, 114 were predicted to be linked through the combination of regulatory networks to basal breast cancer, 48 to luminal and 7 to Her2-positive breast cancer. The reported approach to mining molecular markers is not limited to breast cancer and therefore offers a widely applicable strategy of biomarker mining.

## 1. Introduction

### 1.1. Breast Cancer

Breast cancer covers a diverse range of different diseases [1] and constitutes the second most common type of cancer amongst female patients, after skin cancer [2]. Traditionally, breast cancer is classified according to its histological features and is considered to include at least 11 different histopathological types [3]. The most common type of breast cancer, with 50–80% occurrence, is invasive ductal carcinoma not otherwise specified (IDC NOS), while the other 25% are classified as special types [3]. An alternative classification based on gene expression profiles identifies four molecular types: ER-positive luminal-like, basal-like, ErbB2-positive and normal-like [4,5]. Luminal-like cancer is often divided into luminal A and luminal B subtypes; these will also express combinations of estrogen receptor α (ER) and progesterone receptor (PR) [6]. While both luminal subtypes express similar levels of ER, luminal B tumors overexpress proteins related to proliferation and the cell cycle [7]. Her2-positive breast cancers will overexpress Her2 [8]. The fourth group of cancers, lacking all three established markers, is referred to as triple-negative and accounts for 10–15% of breast cancers. Basal-like breast cancer largely overlaps with triple-negative breast cancer and is named after its common expression of basal cytokeratins [6]. These include keratins 5/6 and 17, which may be expressed in 3–15% of all breast cancers [4].

### 1.2. Traditional Approaches to Screening

The existing Breast Cancer Screening Program in the UK relies on mammography as the main screening modality and is currently exclusive to women aged 50 to 70, although the effectiveness of extending the screening age parameters from 47 to 73 is being investigated [8]. Whilst ample evidence exists supporting mammography as an efficient routine screening approach to finding breast cancer at an early stage, it still yields a substantial number of false-positive tests (over 3% in the UK, [9]) and false-negative tests (one in eight cases [10]), leading to unnecessary additional testing or potential delays in cancer diagnosis respectively. The benefits of mammography-based screenings starting at the earlier age of 40 are further offset by the increased harm from overdiagnosis, overtreatment and the increases in radiation-induced breast cancer [11]. Specificity and sensitivity of cancer detection by mammography alone are not sufficiently high and it is comparable to the levels achieved with clinical breast examination (CBE) as a sole screening modality [12], but the use of CBE in addition to mammography improves both sensitivity and specificity of detection [13]. Traditional X-ray mammography is less sensitive for mucinous, lobular, and rapidly growing cancers [14]. Its relatively low specificity necessitates the use of other imaging modalities such as ultrasonography [15] and magnetic resonance imaging [16], with follow-up examinations and diagnostic evaluation of the suspected or identified tumors [16]. Additional screening modalities are used for higher-risk patients [17], in younger women [18] and in other cases, e.g., recognized symptoms or palpable lesions [19]. However, despite the implementation of regular screenings programs by many developed countries, many breast cancers are being diagnosed at late stages [20]. Combining mammography and tomosynthesis does not improve the effectiveness of screening and does not allow early detection of tumors either [21].

### 1.3. Molecular Approaches to Clinical Diagnostics

Molecular approaches to clinical diagnostics and treatment of breast cancer first came into clinical practice some 20 years ago following the development of the immunohistochemistry (IHC) test for the detection of human epidermal growth factor receptor 2 (HER2/ERBB2/NEU), but the number of validated protein biomarkers in clinical diagnostics remains low. The predictive genetic test for cancer-risk genes *BRCA 1/2* aims to identify germline mutations however, it is limited to the prediction of the likelihood of breast cancer occurrence and its cost-effectiveness remains another issue [22]. Another existing genetic test aims at identifying *PIK3CA* mutations that occur in over 1/3 of breast cancers, most often showing an ER-positive/HER2-negative molecular phenotype and which might indicate a poor response to trastuzumab [23,24]. Elevated serum levels of CEA, CA 15.3/CA 27.29, or CA125 are often found, especially in Her2-positive breast cancer and might indicate the presence of metastatic disease, advanced stages of breast cancer or recurrence respectively. These markers are therefore most suitable for disease monitoring rather than early diagnosis [25,26]. Selectivity of the markers is low; CEA and CA125 are also elevated in liver, lung, breast, ovarian, uterine, cervical, pancreatic and gastrointestinal (GI) cancers, lymphoma and melanoma. CA 15.3 is elevated in lung, pancreatic, ovarian, cervical, prostate, endometrial, bladder, and colon cancers, and other non-cancerous breast lesions; the elevated levels can also be detected in a wide range of non-malignant conditions such as liver cirrhosis, hepatitis, lupus, sarcoid, tuberculosis in pregnancy and lactation [27]. The existing range of plasma, serum or immunohistochemical cancer tests remains limited to a small number of protein targets, unsuitable for mass screening of asymptomatic breast cancer (reviewed in [28,29]). Comprehensive molecular patterns describing distinct breast cancers can be identified using genetic typing and transcriptomics approaches [30]. These and proteomics-based methods, whilst being immensely powerful and remarkably informative research tools, remain largely unsuitable for wider use in clinical diagnostics, which is unsurprising considering the costs [31,32,33]. Biomarkers used currently for clinical diagnosis of breast cancer are reviewed in [28]. There exist a few genetic breast cancer tests to complement traditional pathology testing approaches, but these often rely on the availability of excised cancer tissue samples following surgery, fixed paraffin-embedded cancer samples or biopsies and therefore cannot be used for screening or early diagnostics. There is an apparent lack of validated molecular markers suitable for the minimally invasive or non-invasive detection and molecular diagnosis of asymptomatic breast cancer. Affordable means of mass routine molecular screening remain virtually non-existent. Molecular markers are also needed for the stratification and molecular subtyping of breast cancer. In the absence of suitable molecular tools, pathologic analysis of breast tissue biopsies from identified lesions achieves the highest sensitivity and specificity of detection and remains the key procedure for diagnosis and typing of breast cancers.

### 1.4. Working Hypotheses

**Figure 1:**
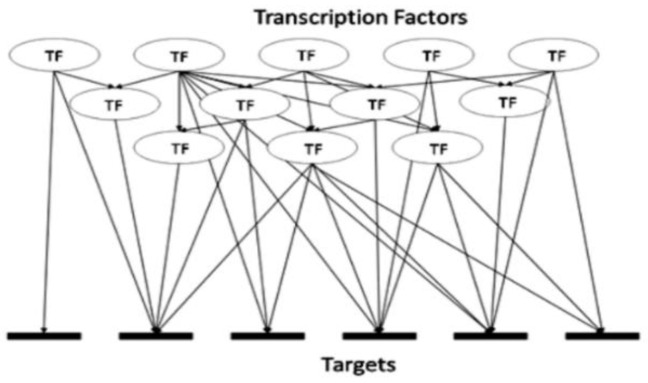
Complexity of transcription factor (TF) hierarchy. A typical range of relationships includes one TF being linked to one target gene, one TF linked to many target genes, many TFs linked to the same one target gene and TF(s) linked to gene(s) encoding TF(s).

There exist a limited number of breast cancer markers, known in the literature to be associated with different molecular subtypes of breast cancer, typical examples are listed in Supplementary Tables S1–S3. There also exists a vast and growing body of knowledge describing gene expression, regulation and function. We set to test whether (1) such knowledge could be used to mine additional and possibly novel biomarkers by using just a few known and proven molecular markers and relying on a ‘guilt by association’ approach and whether (2), such a generic mining approach would yield subtype-specific breast cancer markers, or a ‘generic’ pan-cancer set of markers. To improve the likelihood of reliable predictions we combined three orthogonal data mining techniques. Exploring the transcriptional regulation of genes defines the first dimension of our mining strategy. Any gene is regulated by one or a number of transcription factors (TFs). Detailed information about these TFs may or may not be available for all genes, but a growing number of these have already been described and are obtainable from multiple databases, e.g., TRRUST or TRANSPATH [34,35]. The existing data should therefore allow for the identification of known TFs involved in the regulation of the given genes. That knowledge should also lead to the identification of other genes being regulated by the same TFs, although in some cases it may not be possible to decide whether the connection is inhibitory or excitatory. The approach is exemplified in Figure 1.

Whilst such extrapolation of TF-related connections is expected to yield a vast number of genes potentially related to the initial ‘seed’ genes used, these may not be entirely accurate predictions on their own or be expected to represent complete sets of co-regulated genes. However, with the addition of further independent predictors, the likelihood of identifying genes being genuinely related to the disease and to the initial ‘seed’ markers should increase to allow for distinguishing the new predicted markers from the expected ‘biological noise’. Therefore, we also explored the knowledge of functional biological pathways, as our second independent predictor.

**Figure 2:**
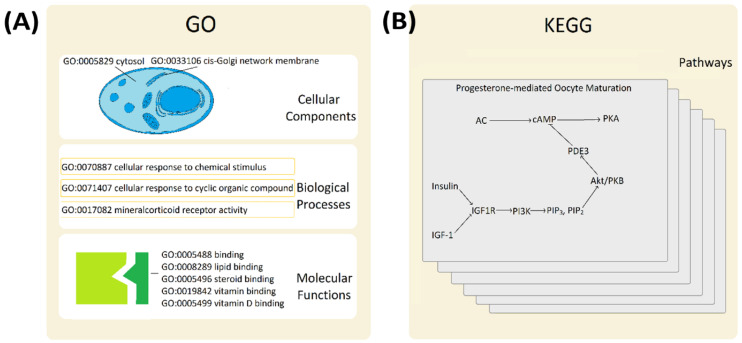
Functional interactions and biological pathways. Panel (**A**). Gene Ontology (GO) database describes three main functional classes: cellular components, biological processes and molecular function. A few typical examples are given to illustrate the variety of classifiers. Panel (**B**). Kyoto Encyclopedia of Genes and Genomes (KEGG) details functional relationships within metabolic pathways, genetic information processing, environmental information processing, cellular process, organismal systems, and human disease pathways. The progesterone-mediated oocyte maturation pathway (hsa04914) is shown as an example of a relevant pathway.

Gene products (functional proteins) do not function in isolation and are often involved in one or many functional pathways (Figure 2). Since changing pathway efficiency or its metabolic flux normally requires simultaneous and coordinated regulation of all proteins involved, identifying such co-involved proteins is likely to yield co-regulated genes. That principle should not be limited to cancer cells readjusting their metabolic fluxes to mitigate the changing energy demands of cancer cells. The same general principle is universally applicable to all complex biological systems from plants to mammals [36,37,38,39]. Such functional regulation might be further complicated in cases of branched pathways. However, whilst modelling complex pathways is far beyond the focus of this investigation, the use of existing biological pathway network information does provide a useful shortcut for identifying smaller gene sets of functionally related gene products, which are likely to be similarly regulated at the gene expression level.

**Figure 3:**
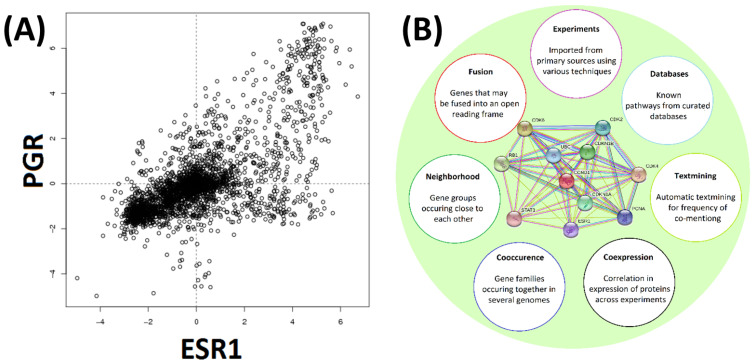
Gene expression and protein–protein interaction. Panel (**A**). COXPRESdb uses experimentally determined expression data to identify frequently co-expressed genes. A typical example is shown. Expression levels of *ESR1* and *PGR* are plotted along the horizontal and vertical axes respectively. Concentration values are shown as Log2 relative averaged expression levels of the genes. Panel (**B**). The STRING tool conveniently combines analysis of expression, co-occurrence, neighborhood gene analysis, discovery of fused genes, published experimental evidence and database occurrences. A typical example output for CCND1 is shown (panel center). Differently colored lines between proteins indicate the type of evidence used. Co-expression was relied upon in the current investigation.

The third independent predictor comes from gene clustering analyses based on co-expressions data (Figure 3). Here, an insight into the expression of some genes may be gained from the known expression traits for ‘co-expressed’ genes and assuming that their expression follows the same pattern [40]. None of these three predictors would be entirely accurate, which is especially true for the predictions of disease association from co-expression networks; it is safe to expect a high false-positive rate. All such predictions are also likely to be incomplete and it is reasonable to expect a high false-negative rate as well. However, these three mining approaches provide the three largely independent prediction methods. Therefore, combing together all three approaches and using them with well-characterized cancer-related proteins should achieve better accuracy, reduce the number of predicted false positives and should help with identifying relevant cancer networks starting with just a few initial ‘seed’ genes. We set to test such our data mining approach with the aim to generate an expanded set of genes (and their products) which could potentially be the most likely candidates for cancer targets or cancer markers. One particularly interesting question was whether such a ‘guilt by association’ mining approach would remain biased towards generic pan-cancer networks and generic markers or whether such bias could be avoided to favor subtype-specific breast cancer markers.

## 2. Materials and Methods

### 2.1. Selection of Known Markers for Use as Seeds in the Subsequent Analyses

Known markers used as ‘seeds’ in the subsequent pathway and network analyses, were identified from literature to represent three major subtypes of breast cancer. In this case, ESR1, ESR2, PGR, CCND1, FOXA1, GATA3, KRT18, KRT8, LAPTM4B, SLC39A6, SQLE, TFF3, and XBP1 were chosen for luminal breast cancer (Table S1). ERBB2 and GRB7 were selected for Her2-positive breast cancer (Table S2), while KRT5, CDH3, ID4, FABP7, KRT17, TRIM29, LAMC2 and ITGB4 were used to search for an expanded set of basal type-specific breast cancer markers (Table S3).

### 2.2. Transcriptional Networks

Two separate tools were used to identify potential marker genes based on their transcriptional regulatory relationships with the known ‘seed’ markers. Both TRANSPATH [41] and TRRUST [42] tools were used with default settings, the searches were limited to *Homo sapiens*. In both cases, TFs known to regulate given genes were identified first, and then used to compile lists of known target genes for all these TFs, from both databases, to ensure maximum coverage. In all cases, the annotation terms which suggested transcriptional regulatory relationships were relied upon to select genes. Other terms suggesting transcriptional regulation were considered for gene inclusion, other terms such as molecular interactions were not considered and those genes were not included in further analysis. In both tools, genes shown to regulate a known marker were considered further as potential biomarkers. The genes targeted by a known marker, if it was a TF, were also considered for further analysis. When combining the data from the two different searches, a candidate biomarker was considered for further analysis if identified in at least one of the searches.

### 2.3. Biological Pathways

Two separate tools were used to identify genes based on their pathway associations: Gene Ontology (GO) [43] and Kyoto Encyclopedia of Genes and Genomes (KEGG) [44]. The annotation categories (molecular functions and biological processes) were searched and identified for the known ‘seed’ markers using GO [45]. Lists of all *Homo sapiens* genes/proteins with annotations matching those of the known markers were then obtained from the National Centre for Biotechnology Information (NCBI) platform [46]. The KEGG database entries for the known markers were searched to identify their pathway entries [47]. Lists of all other *H. sapiens* genes/proteins associated with the identified KEGG pathways were obtained from the relevant pathway pages [47]. Only molecular functions and biological processes were considered. When combining the data from the two different searches, a candidate biomarker was considered for further analysis if identified in at least one of the searches.

### 2.4. Gene Co-Expression and Protein–Protein Interactions

The COXPRESdb resource [48] was used to identify co-expressed genes and the STRING resource [49] was used to identify interacting proteins. Default settings were used with the COXPRESdb tool [50] and 2000 co-expressed genes (the maximum allowed) were considered. Only human pathways data were considered. The STRING tool [51] was used to find proteins that had been experimentally determined to interact with the given markers. The minimum required interaction score was adjusted to low confidence (0.150) and the maximum number of interactions to show was changed to 100 in the first shell [51].

### 2.5. The Overall Prediction Strategy

**Figure 4:**
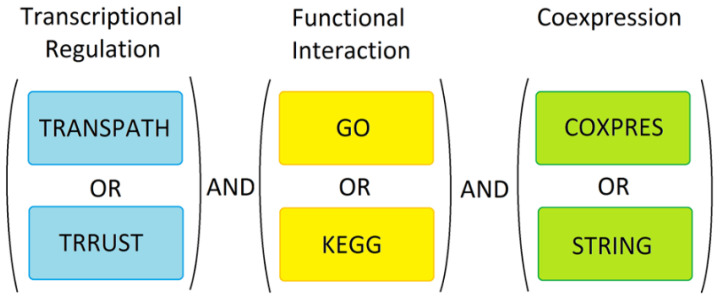
Marker discovery strategy. The known markers were used for analysis in TRANSPATH, TRRUST, GO, KEGG, COXPRESdb and STRING. In order to select potential new markers, we combined the three data sets as shown. Genes/proteins were only considered further if they were transcriptionally regulated, functionally related and were experimentally proven to be co-expressed or known to physically interact with the known markers.

Special care was taken to account for the incompleteness of databases and potential bias due to some of the TFs, pathways or co-expression networks having been studied better than others. Two separate tools were used for each of the three independent prediction approaches (as outlined above) and at each individual search stage, identical weights were given to all predicted candidate genes, irrespective of how many times any gene/protein was found or how many TFs, pathways or co-expression networks were known to be involved in each gene’s regulation, thus resulting in a binary selection outcome for all genes at individual search stages. Whether a gene was found using only TRRUST or TRANSPATH or whether it was found using both did not affect the inclusion of such gene in further analyses. This was done in order to compensate for deficits in either tool, as no database was assumed to be complete. The same applied to GO and KEGG, and COXPRESdb and STRING. None of the tools or databases used were considered to be entirely accurate or complete. However, only genes/proteins identified in all three analyses were considered predictive markers. Therefore, to be considered for further analysis a candidate biomarker had to be transcriptionally regulated as, functionally interact with, be co-expressed with, or interact with at least one of the known markers used as ‘seeds’ in our search. Where necessary, the DAVID conversion tool [52] was used to convert proteins and genes to common IDs to allow the combining of the different candidate biomarkers. The overall search strategy is summarized in Figure 4. At all stages, the ‘seed’ genes belonging to each one of the three different major groups of breast cancer (Tables S1–S3) were processed and analyzed separately from the ‘seed’ genes belonging to the other two groups (i.e., basal, luminal, Her2-positive).

### 2.6. Analysis of Protein Targeting and Further Marker Validation

Proteins encoded by the identified genes were further checked for their cellular location, in particular, whether or not they were secreted, as this would make them more useful as markers for early or minimally invasive detection. To identify secreted proteins, all sequences were checked for the presence of signal peptides and transmembrane domains. Protein sequences were obtained from UniProt [53] for each of the proteins. The sequences were analyzed for signal peptides and transmembrane domains using the Phobius tool [54]. The selected output format was changed to short, otherwise default, settings. A protein was deemed to be secreted if the sequence encoded a signal peptide but did not code for a transmembrane domain.

To further independently validate the predictions made, we analyzed transcription levels of the predicted marker genes. mRNA expression data representing human breast cancer and matching non-cancer tissues were obtained from the NCBI Gene Expression Omnibus (GEO), Affymetrix human genome microarray data set GSE124646 [55], which has undergone additional quality control and regression fitting prior to the analysis, as described in [56]. Log-transformed transcription level changes in breast cancer tissues compared to matching normal tissue biopsies were considered significant if *p* < 0.05. To discover over-represented biological pathways, the gene ontology identifiers assigned to the identified significantly upregulated genes were searched for and compared to the gene ontology information assigned to the entire gene set from the Affymetrix Human Genome U133A Array dataset used for mRNA expression analysis [55,57].

## 3. Results

### 3.1. Analysis of Transcriptional Networks Yields Potentially Co-Regulated Genes

**Figure 5:**
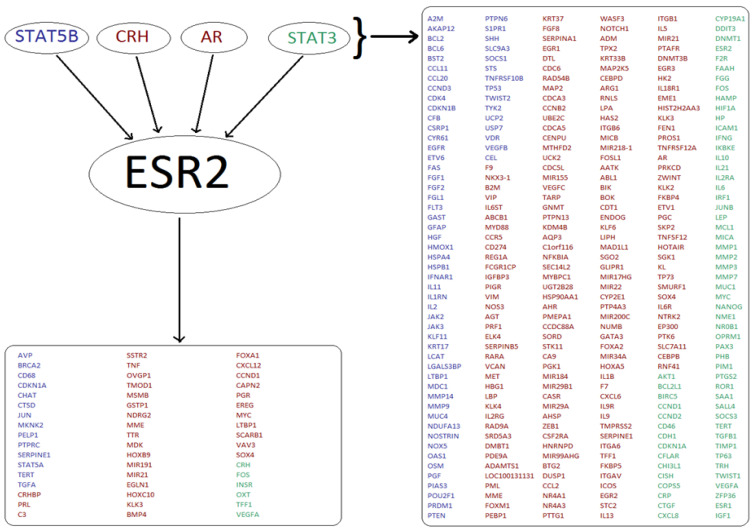
Analysis of TF regulatory networks yields many potentially co-regulated genes. A typical result is shown for *ESR2*. Four TFs (STAT5B, CRH, AR, STAT3) which regulate *ESR2* expression also regulate 306 other genes (listed on the right). The *ESR2* gene encodes Estrogen Receptor 2, a nuclear hormone receptor which upon binding estrogen can activate the expression of genes containing estrogen response elements (ERE) (51 genes shown in the bottom left box). Results generated using the TRANSPATH tool are shown in red, whilst genes identified using the TRRUST tool are shown in blue. Targets found using both tools are shown in green.

Interrogation of TF databases identified many potentially co-regulated genes. A typical outcome of one such individual search is illustrated for the ESR2 protein, one out of the 23 original ‘seed’ markers. The search illustrated in Figure 5 identified four known TFs, which in turn pointed toward 306 potentially co-regulated genes. In addition to these, 51 genes are known to also be directly regulated by ESR2. Interestingly, but not unexpectedly, the two tools used (TRANSPATH and TRRUST) generated different sets of TFs even where the same ‘seed’ genes were used and these often resulted in the very limited overlap. In the example used, the two pools of the predicted ESR2-related genes from TRANSPATH and TRRUST searches overlapped by less than 50%. The outcome was similar in all the remaining 22 ‘seed’ marker searches. Overall, 2338 different genes were identified as being co-regulated with at least one of the 13 ‘seed’ markers for luminal breast cancer. In the analysis of two Her2-positive breast cancer markers, 571 co-regulated genes were identified in TRANSPATH and TRRUST combined, with no data available in either of the databases for *GRB7*. 1465 different genes were predicted to be co-regulated at the transcriptional level with the 8 markers representing basal breast cancer by the two tools combined.

### 3.2. Analysis of Biological Pathways Identifies Potentially Functionally Related Genes

**Figure 6:**
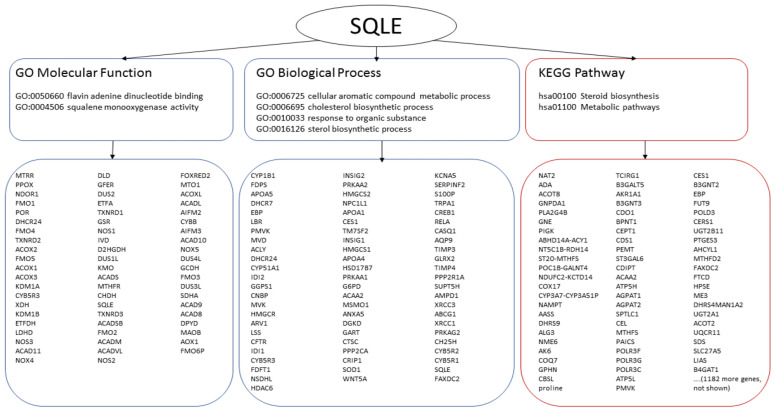
Analysis of pathways and biological processes. SQLE is used to illustrate a typical output. SQLE is annotated with two molecular functions, four biological processes and two pathways. The data obtained from GO are encircled with blue lines (left and middle), the data obtained from KEGG are encircled with red lines (right). Interrogation of the functional groups yields many potentially co-involved genes/proteins.

Interrogation of biological pathway databases also yielded many potentially co-involved proteins. A typical outcome of one such search for SQLE, one out of the 23 ‘seed’ markers, is summarized in Figure 6. In total, 11,621 different proteins were found to be associated with the 13 markers of luminal breast cancer in either the GO or KEGG database, 6861 proteins were predicted for two Her2-positive breast cancer and 7910 proteins were found to be potentially co-involved with the eight basal breast cancer markers.

### 3.3. Exploring Gene Co-Expression and Protein Interaction Data

**Figure 7:**
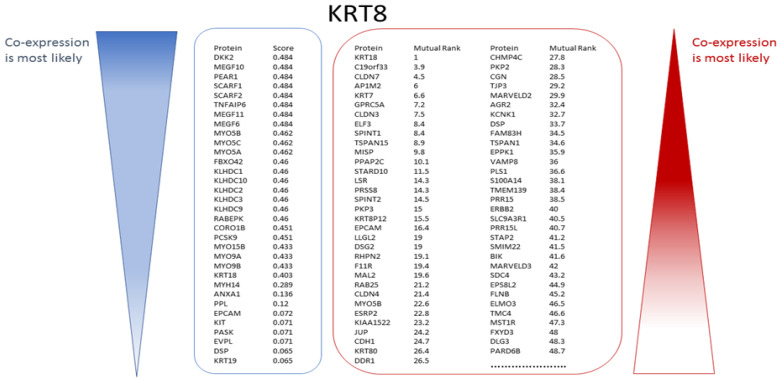
Many candidate genes/proteins can be identified from experimental co-expression (illustrated for KRT8). STRING yielded 33 proteins co-expressed with KRT8. The proteins are ranked in descending order of their unit-less co-expression scores (indicator blue bar, left). COXPRESdb always yields a list of 2000 potentially co-expressed genes/proteins ranked in the order of their Mutual Rank (MR) where the value of 1 indicates the strongest likelihood of co-expression. Of these COXPRESdb gene results, the 65 top ranked co-expressed genes/proteins are shown (indicator red bar, right).

COXPRESdb and STRING were used to identify proteins that have been experimentally determined to be co-expressed with the known markers. A typical outcome of one such search for KRT8, another of the 23 ‘seed’ markers, is illustrated in Figure 7. The search using the STRING tool yielded 33 proteins and the search with COXPRESdb yielded a 2000 proteins-long ranked list, of which the top 65 are shown in Figure 7. In total 11,147 different genes were predicted to be potentially co-expressed with at least one of the 13 markers for luminal breast cancer. A total of 3127 different genes were identified as co-expressed with at least one of the two Her2-positive breast cancer markers and 7918 different genes were found starting from the eight markers for basal breast cancer.

### 3.4. Exploring Gene Co-Expression and Protein Interaction Data

Overall, having started with just a few genes/proteins known to be associated with breast cancer and having explored their transcriptional co-regulation, co-expression and their involvement in the same functional pathways, 459 potential biomarker genes of breast cancer were shortlisted following the analysis of the 13 ‘seed’ markers of luminal type breast cancer. The 459 proteins encoded by these genes were further filtered for transmembrane domains and signal peptides and 317 were found to have neither a signal peptide nor a transmembrane domain, 43 had at least one transmembrane domain and a signal peptide, 51 had a transmembrane domain and no signal peptide and 48 proteins were found to have a signal peptide but no transmembrane domain, indicating extracellular space as the most likely targeting destination. One of these genes/proteins, the trefoil factor 3 (TFF3), was part of the original set of ‘seed’ cancer markers used. Similarly, having started with just two ‘seed’ markers of Her2-positive breast cancer, 66 potential biomarker genes of breast cancer were shortlisted.

**Figure 8:**
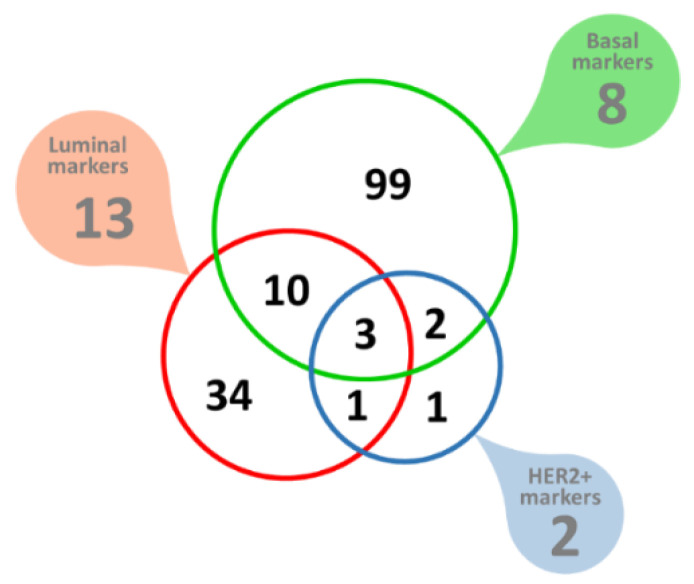
Molecular markers of breast cancer. Filled callout shapes indicate numbers of the original ‘seed’ markers used. The color-coded Venn diagram shows the predicted breast cancer biomarkers, all of which are extracellularly targeted proteins.

Of these Her2-positive breast cancer potential biomarkers, seven proteins were found to have a signal peptide but no transmembrane domain, indicating extracellular space as the most likely targeting destination. The analysis of eight markers of basal breast cancer yielded 520 potential markers, of which 114 had a signal peptide but no transmembrane domain, indicating extracellular space as the most likely targeting destination. One of these, the Laminin subunit γ-2 (LAMC2) was part of the original set of the eight ‘seed’ cancer markers used. There was a small degree of overlap between the potential markers derived from luminal, basal, or Her2-positive marker ‘seeds’, most likely indicating a degree of commonality involved in transcription, regulation and biological pathways between the three distinct breast cancer types explored, but many of the predicted marker genes remained limited to one of the three separate breast cancer types tested, summarized in Figure 8. In total, 150 novel extracellularly targeted breast cancer markers were predicted.

**Figure 9:**
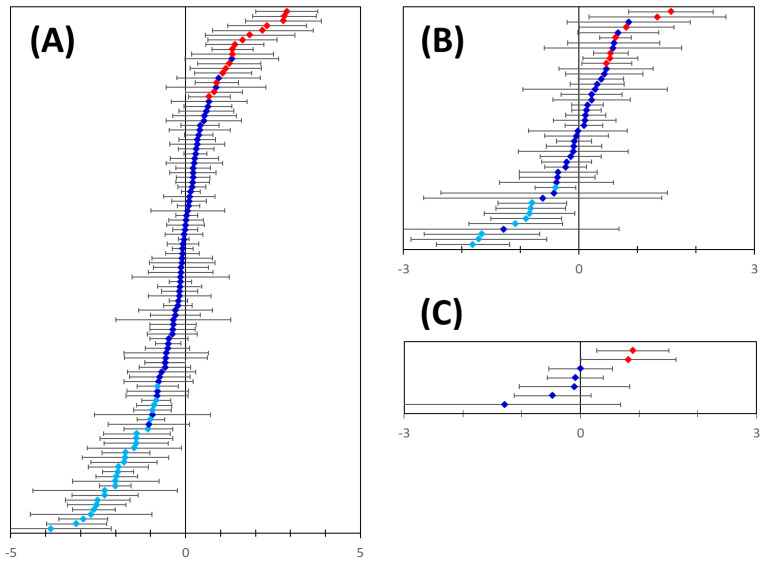
Differential mRNA expression of the predicted genes in breast cancer. Panel (**A**): Microarray gene expression analysis data were available for 110 out of the 114 predicted basal breast cancer markers. 16 out of the 110 transcripts were significantly upregulated (red symbols) and 29 were significantly downregulated (light blue symbols) in breast cancer samples. Panel (**B**): Microarray gene expression analysis data were available for 46 out of the 48 predicted luminal breast cancer markers. 7 out of the 46 transcripts were significantly upregulated (red symbols) and 9 were significantly downregulated (light blue symbols) in breast cancer samples. Panel (**C**): 2 out of the 7 transcripts predicted for Her2-positive breast cancer were significantly upregulated (red symbols) in breast cancer samples. In all panels error bars indicate confidence intervals (*n* = 10, *p* = 0.05).

Our data mining approach has not discriminated between up- or down-regulated proteins. All the analyses so far assumed and aimed at identifying co-regulated genes that included both up- and down-, strongly and weakly regulated genes/proteins. This is to a large degree the result of the complexity of expression regulation by TFs and the incompleteness of TF databases. Therefore, we applied one additional check to (1) validate our predictions and (2) estimate the nature and the degree of differential regulation. We relied on publicly available and quality-controlled gene expression data [55,56] to get independent evidence of differential gene expression for the 150 putative markers identified in this work. Expression data were available for 110 out of the 114 newly predicted basal breast cancer markers (of which 99 markers were limited to basal type only, Figure 8. Of these, 16 were found to be significantly upregulated and 29 significantly downregulated in breast cancer tissues (Table 1 and Table 2, Figure 9A). Expression data were also available for 46 out of the 48 newly predicted luminal breast cancer markers (of which 34 were limited to luminal type only, Figure 8. Of these, seven were found to be significantly upregulated and nine were significantly downregulated in breast cancer tissues (Figure 9B, Table 1 and Table 2). A similar analysis of the newly predicted Her2-positive breast cancer markers yielded two significantly upregulated transcripts (Figure 9C, Table 1). Altogether, out of the 150 predicted extracellularly targeted protein markers 58 were confirmed at gene expression level to be significantly changed in the pool of breast cancer samples included in the analysis. Of the 58 dysregulated transcripts 21 transcripts showed significant upregulation (Table 1) and 37 markers showed significantly reduced transcription levels (Table 2). Two of the 21 upregulated markers have not been linked to breast cancer in the past, whilst the rest represent proteins strongly linked to breast or other cancers, based on published evidence.

Furthermore, all but four of these genes appear to be subtype-specific breast cancer markers (Table 1 and Table 2). Whilst accurate quantification of gene expression of the individual markers is outside the scope of this study, consistency between our predictions and the microarray data validates our approach to marker discovery and proves the hypothesis that cancer markers could indeed be predicted starting with an only a small number of known markers and utilizing the existing knowledge of gene and protein networks. Our results also indicate that extracellularly targeted cancer-associated proteins may be predicted starting with intracellular disease-associated genes/proteins.

We also checked which biological pathways, if any, were over-represented among these 58 predicted and validated genes. Table 3 illustrates the top 20 over-represented biological pathways as defined by their gene ontology. The five most over-represented pathways are collagen catabolic process (GO: 0030574), extracellular matrix disassembly (GO: 0022617), platelet degranulation (GO: 0002576), positive regulation of cell migration (GO: 0030335) and extracellular matrix organization (GO: 0030198)—all being highly relevant to cancer, which further confirms the validity of our predictions. As expected, the vast majority of the predicted and validated genes (55 out of 58) were confirmed as belonging to extracellular regions, following a similar analysis of over-represented cellular components among the 58 identified genes (Table 4). In total, 21 of the 58 proposed markers, which have been confirmed as significantly over-expressed at the transcription level (Table 2). In total, 19 of these have been associated with multiple malignancies including breast cancer, based on the current literature, but no direct evidence exists though to link the remaining two of the 21 markers (EDEM2 and IL18BP) to breast cancer, making these the most promising novel breast cancer markers identified here. It is also quite possible that more than just 58 of the predicted 150 markers could be confirmed at gene expression level if a larger patient cohort was used with a wider range of cancer types and cancer stages.

## 4. Discussion

Molecular biomarkers play an ever-increasing role in diagnosis, risk stratification, prognosis and predicting the outcome of disease or treatments [58,59,60]. Integration of multiple molecular approaches with pathological and clinical outcomes justifies new classifications, defines multiple distinct cancer subtypes and genetic risk scores [61,62,63]. Traditional serologic testing used for diagnosis and management of breast cancer relies on carcinoembryonic antigen (CEA) for cancer detection and monitoring recurrence, carbohydrate antigen 15.3 (CA15.3) for monitoring metastatic (stage 4) breast cancer and its response to treatment and carbohydrate antigen 27.29 (CA27.29) for predicting recurrence. None of these tests possess high sensitivity or specificity or are suitable for routine cancer screening applicators. Multiple other conditions may cause an increased markers’ concentration, such as in colon, liver, lung, pancreatic and prostate cancers, liver cirrhosis, some infections, endometriosis, lupus, smoking or pregnancy, making any diagnosis inaccurate.

Early efforts to develop molecular screening tools for the detection and characterization of breast cancer date back to early the 2000s [4,5]. Nowadays, a few clinical gene expression assays are available to assist with molecular classification of breast cancer, the assignment of breast cancer therapy and for predicting metastases-free and overall survival. These include the Prosigna breast cancer prognostic assay (the PAM50 test) for quantifying the expression of 50 genes in early stage, hormone-receptor-positive breast cancer and prediction of tumor metastasis [64,65]. The MammaPrint and BluePrint assays (Agendia) for quantifying the expression levels of 70 and 80 genes, respectively, in the early stage breast cancer for guiding chemotherapy, estimating the risk of recurrence [66] and for molecular classification of breast cancer [67] and the breast cancer index (BCI) PCR-based 7-gene prognostic assay, to predict response to Tamoxifen and clinical outcomes including distant recurrence in ER-positive cases [68,69]. Oncotype DX is another PCR-based test of a panel of 21 genes for calculating a prognostic recurrence score and guiding chemotherapy in ER-positive, node-negative breast cancer [70]. One other molecular test is MapquantDx genomic grade index (GGI) which uses a 97-gene expression panel to define histological grade and predict the risk of recurrence [71]. All these tests could be used with formalin fixed paraffin embedded tissues, with MammaPrint and GGI also benefiting from using fresh tissue, such as fine-needle aspiration biopsies. All the above gene panels rely on the excised tumor tissues for extracting the transcribed genes. None of these tests are designed to be used with blood or serum which makes all such tests unsuitable for cancer screening purposes. Most of these gene panel sets were developed using either retrospective analyses of formalin fixed paraffin embedded materials or within a few exceptions largely high-grade tumors, which is a typical experimental approach and a limitation of such retrospective analyses. However, genes upregulated in higher grade tumors may be quite different from those affected at the very early stages of tumorigenesis. IHC remains the method of choice when testing for HER2 as well as for determining hormone receptor status, e.g., with the HercepTest (Daco), Insight Dx Mammostrat Plus (Clarient Diagnostic) or PATHWAY (Ventana Medical Systems) [72]. Other methods rely on testing gene copy numbers using FISH, e.g., PathVysion Her-2 DNA Probe Kit (Abbott Molecular) [73], CISH, e.g., Her2 CISH pharmDx kit (Dako) or by Gene Expression Tests, e.g., TARGETPRINT (Agendia) to name a few. Oestrogen and progesterone receptors in breast cancers are also assessed by IHC from formaldehyde-fixed paraffin-embedded tissues or needle biopsies (e.g., ER/PR pharmDx assay kit, Dako). Another protein marker assay tests for invasion and metastasis markers urokinase-type plasminogen activator (uPA)/plasminogen activator inhibitor-1 (PAI-1), using enzyme-linked immunosorbent assay (ELISA) to guide treatment in node-negative (N0) breast cancer. Pathology reports routinely include information about HER2 status. The reliance on the tumor tissues as the source of test materials precludes all such methods from wider largely healthy population screening applications. Traditional marker discovery methods are also not entirely suitable for the discovery of very early markers of cancer development—where no surgically removed material is available and when the test population would be largely healthy. Early and minimally invasive detection of breast cancer remains among key unmet needs and research gaps in the Fight against breast cancer [74,75]. Mammography currently remains the most common way of screening the population for breast cancer [76], including the USA and UK [77].

Whilst a few breast cancer-related genes and proteins have been identified and characterized over the course of the last few decades, most of these are late-stage markers and often intracellular proteins identified in surgically removed tissues often representing late-stage tumors. Tables S1–S3 list several such known markers. We decided to combine these with the knowledge of transcriptional, biochemical and functional protein networks aiming to (1) test an alternative approach for identifying and expanding the range of potential molecular markers of breast cancer by using the “guilt by association” approach and (2), to identify among many potential markers, those gene products (proteins) which could potentially be detected outside the tumor cells, for example in blood or other physiological fluids.

The expression level of a gene can be regulated by one or more TFs, which usually target more than one gene. Therefore, it should be possible to identify some, if not all of the co-regulated genes. However, none of the existing databases may have complete and accurate information relating to the multitude of associated complex transcription networks. We therefore used two different databases of known TFs (TRANSPATH [35] and TRRUST [34]), but refrained from using prediction tools, e.g., [78], to ensure maximum coverage and reproducibility.

Biological pathways represent the molecular-level pathways by which a cellular process such as metabolism or a disease process such as cancer can occur. Each individual pathway describes various mechanisms that lead to overall cell function, allowing the identification of the different genes involved within the specific overall function of the pathway in question [79]. Altered expression levels of individual genes within a specific pathway is seldom singular, with many other genes within the pathway undergoing similar regulatory trends and showing concerted changes to their expression levels to maintain pathway metabolic flux. Knowledge of some of the disease-related genes may therefore lead to the discovery of additional related genes by exploring functional gene and protein networks. We used two different resources (Gene Ontology [43] and KEGG [44]) to ensure better coverage and we did not prioritize gene candidates based on the frequency of their appearance at this stage, to avoid bias due to the gaps in data coverage or due to excessive coverage of some popular biological pathways and traits.

Similar co-regulation may also be expected of proteins that physically interact with each other. The need to optimize the functionality of protein–protein interactions is likely to lead to the interacting proteins being similarly regulated, co-activated or co-expressed. The two different resources used to explore this phenomenon (and to achieve maximum coverage) were COXPRESdb [48] and STRING [49].

The analyses of transcriptional regulation, functional or physical interaction and co-expression represent orthogonal prediction approaches, therefore any false positives predicted with one tool are likely to have been filtered out by the other approaches. Combining these independent predictions, as outlined in Figure 4, yielded many potential markers expected to be dysregulated in breast cancer, in a fashion similar to the few known breast cancer markers used as ‘seed’ genes in our strategy. Of these we focused on the transcripts which encoded proteins with signal peptides but without TM domains, thus selecting proteins destined for extracellular space. Our data mining approach yielded 150 potential extracellularly targeted breast cancer-associated proteins (Table S4) starting with just 23 initial ‘seed’ markers, of which only two were known to be extracellular targeted proteins (LAMC2 and TFF3). Ultimately, LAMC2 and TFF3 were also found among the 150 potential markers identified here, as expected. The remaining 148 of the putative extracellular markers identified here were discovered using a pool of largely intracellular ‘seed’ marker proteins, thus confirming our initial expectations. Many of such identified markers represent known extracellular matrix proteins and metalloproteinases (Table S4). Some of the predicted 150 proteins are known from published literature to have some association with other cancers or diseases, including breast cancer in a few cases (Table S5), which strongly supports our approach to mining the potential biomarkers. Such findings indicate that our search strategy has been successful in its ability to independently identify relevant disease-associated proteins. However, since the majority of these proteins are targeted to extracellular locations, many have escaped detection in proteomics-driven studies that typically focus on the solid tumor tissues and hence membrane-associated or intracellular proteins. Being extracellularly targeted and often, but not always, associated with cell membranes, few if any of these have been considered suitable cancer targets in the past. Furthermore, out of the 150 potential markers identified in this study, a few proteins Defensin (DEFA4), α-2-macroglobulin receptor-associated protein (LRPAP1), Interleukin-18-binding protein (IL18BP), Interleukin-17D (IL17D) and ER degradation-enhancing α-mannosidase-like protein 2 (EDEM2) are not currently linked specifically to breast cancer and may therefore represent completely new markers of breast cancer potentially suitable for population screening applications. The predicted putative marker proteins EDEM2 and IL18BP are especially interesting, because their genes were found to be significantly upregulated in all breast cancer microarrays included in the analysis (Table 1).

The three proteins found in all three independent analyses for the different subtypes of breast cancer were EPO, VEGFA and KLK3. These are highly interesting as they represent single proteins that could potentially be used to detect all subtypes of breast cancer as well as other cancers. EPO was traditionally associated with its effects on red blood cells, specifically its role in erythropoiesis [80]. It also has a wide range of effects on cancer cells, including an anti-apoptotic effect [80]. Due to its role in many cancers, EPO could be useful in the detection of cancer in general. VEGFA also plays a role in various cancer types and angiogenesis in general [81]. It also stimulates the migration of tumor cells [81]. VEGFA is considered to be not only a marker but also a potential target for the prevention of angiogenesis, which only involves a few proteins [81]. KLK3 has long been used as a marker for prostate cancer [82]. Furthermore, the promoter and enhancer regions of KLK3 have been shown to be mutated in breast cancer [82]. This suggests the role of KLK3 in several types of cancer, making it a potential diagnostic marker for cancer in general.

As our approach to marker mining does not discriminate between up- or down-regulated genes and employed no means of predicting the degree of gene expression dysregulation, we did not expect all the 150 genes to be up-regulated. Considering the complexity of regulatory networks, we did not expect a simple binary up/down expression regulation outcome either but, we tested our predictions using microarray gene expression data available publicly from [83] and further quality controlled as described in [56]. 58 of the proposed 150 markers (37 downregulated and 21 upregulated, totaling just over 40%) were significantly dysregulated at the transcription level (Table 1 and Table 2 and Table S4). Whilst the gene expression analysis was based on a limited scale single microarray study [55], that was justified due to the high quality of expression data [56]), the gene expression analysis has validated our data mining strategy.

## 5. Conclusions

The main challenges underlying the key hypotheses behind this research were to check if cancer markers could be mined based on partial knowledge of transcriptional and functional protein networks and using few known cancer-associated genes/proteins to guide the search. The second challenge was to check whether genes encoding extracellular protein markers could be predicted using known marker genes typically encoding intracellular proteins. This second challenge was especially important because the availability of extracellular molecules should greatly facilitate early cancer marker discovery. We addressed both points and showed that our approach to mining cancer markers can predict meaningful putative markers, including genes encoding extracellular proteins, some of which are secreted and therefore could potentially be detected in biological fluids such as blood or urine. Unlike many traditional markers and especially cancer targets, the predicted proteins lack membrane domains and with the exception of matrix proteins are not confined to intracellular or membrane compartments. Such protein markers could potentially be detected using minimally invasive or non-invasive methods (urine, blood or other physiological liquid samples) rather than needle biopsies. The predicted markers therefore represent an expanded set of molecular tools potentially suitable for population screening applications using liquid biopsies of cancer. The reported biomarker mining approach for mining molecular markers is not limited to breast cancer and therefore offers a widely applicable strategy for biomarker discovery.

## Figures and Tables

**Table 1:** Predicted extracellularly targeted genes/proteins significantly upregulated in breast cancer.

Gene	UniProt ID	Protein Name	HG-U133A ^1^	B ^2^	L ^3^	H2 ^4^
BGN	P21810	Biglycan	201262_s_at	X		
CEMIP	Q8WUJ3	Cell migration-inducing and hyaluronan-binding protein	212942_s_at	X		
CXCL10	P02778	C-X-C motif chemokine 10	204533_at	X		
CXCL8	P10145	Interleukin-8	211506_s_at	X		
HPSE	Q9Y251	Heparanase	219403_s_at	X		
INHBA	P08476	Inhibin β A chain	204926_at	X		
MMP1	P03956	Interstitial collagenase	204475_at	X		
MMP11	P24347	Stromelysin-3	203878_s_at	X		
MMP12	P39900	Macrophage metalloelastase	204580_at	X		
MMP13	P45452	Collagenase 3	205959_at	X		
MMP9	P14780	Matrix metalloproteinase-9	203936_s_at	X		
PLAU	P00749	Urokinase-type plasminogen activator	205479_s_at	X		
PLAUR	Q03405	Urokinase plasminogen activator surface receptor	214866_at	X		
FN1	P02751	Fibronectin	214702_at	X		X
VEGFA	P15692	Vascular endothelial growth factor A	210512_s_at	X	X	X
COL1A2	P08123	Collagen α-2(I) chain	202404_s_at	X	X	
CTSD	P07339	Cathepsin D	200766_at		X	
EDEM2 ^5^	Q9BV94	ER degradation-enhancing α-mannosidase-like protein 2	218282_at		X	
HSPA5	P11021	78 kDa glucose-regulated protein	211936_at		X	
IFNG	P01579	Interferon γ	210354_at		X	
IL18BP ^5^	O95998	Interleukin-18-binding protein	219323_s_at		X	

^1^ Affymetrix Human Genome U133A Array; ^2^ Basal-like breast cancer markers; ^3^ Luminal-like breast cancer markers; ^4^ Her2-positive breast cancer markers; ^5^ No prior publications on the role in breast cancer or other cancers.

**Table 2:** Predicted extracellularly targeted genes/proteins significantly downregulated in breast cancer.

Gene	UniProt ID	Protein Name	HG-U133A ^1^	B ^2^	L ^3^	H2 ^4^
ADAMTS1	Q9UHI8	Disintegrin and metalloproteinase with thrombospondin motifs 1	222162_s_at	X		
BMP2	P12643	Bone morphogenetic protein 2	205289_at	X		
BMP4	P12644	Bone morphogenetic protein 4	211518_s_at	X		
CHRDL1	Q9BU40	Chordin-like protein 1	209763_at	X		
CTGF	P29279	Connective tissue growth factor	209101_at	X		
CYR61	O00622	Protein CYR61	201289_at	X		
DCN	P07585	Decorin	209335_at	X		
EDN1	P05305	Endothelin-1	218995_s_at	X		
FBLN1	P23142	Fibulin-1	201787_at	X		
FIGF	O43915	Vascular endothelial growth factor D	206742_at	X		
FST	P19883	Follistatin	207345_at	X		
IGF1	P05019	Insulin-like growth factor I	209540_at	X		
IGFBP3	P17936	Insulin-like growth factor-binding protein 3	210095_s_at	X		
LAMC2	Q13753	Laminin subunit γ-2	202267_at	X		
LEP	P41159	Leptin	207092_at	X		
LPL	P06858	Lipoprotein lipase	203549_s_at	X		
LTF	P02788	Lactotransferrin	202018_s_at	X		
LUM	P51884	Lumican	201744_s_at	X		
MFGE8	Q08431	Lactadherin	210605_s_at	X		
NID1	P14543	Nidogen-1	202007_at	X		
OGN	P20774	Mimecan	218730_s_at	X		
PDGFD	Q9GZP0	Platelet-derived growth factor D	219304_s_at	X		
PENK	P01210	Proenkephalin-A	213791_at	X		
PROS1	P07225	Vitamin K-dependent protein S	207808_s_at	X		
PTGDS	P41222	Prostaglandin-H2 D-isomerase	211663_x_at	X		
PTGS2	P35354	Prostaglandin G/H synthase 2	204748_at	X		
RELN	P78509	Reelin	205923_at	X		
SOD3	P08294	Extracellular superoxide dismutase	205236_x_at	X		
PDGFA	P04085	Platelet-derived growth factor subunit A	205463_s_at	X	X	
ANG	P03950	Angiogenin	205141_at		X	
C1S	P09871	Complement C1s subcomponent	208747_s_at		X	
CD59	P13987	CD59 glycoprotein	212463_at		X	
KLK1	P06870	Kallikrein-1	216699_s_at		X	
PTHLH	P12272	Parathyroid hormone-related protein	210355_at		X	
SLPI	P03973	Antileukoproteinase	203021_at		X	
SPARCL1	Q14515	SPARC-like protein 1	200795_at		X	
TIMP3	P35625	Metalloproteinase inhibitor 3	201150_s_at		X	

^1^ Affymetrix Human Genome U133A Array; ^2^ Basal-like breast cancer markers; ^3^ Luminal-like breast cancer markers; ^4^ No newly identified Her2-positive breast cancer markers were significantly downregulated.

**Table 3:** GEO biological processes over-represented among the 58 newly identified markers significantly dysregulated in breast cancer.

GEO Gene Ontology Biological Process ^1^		Counts Observed ^2^	Enrichment		*p* Value
			Fold Difference over the Expected ^3^	Rank ^4^	
GO: 0030198	extracellular matrix organization	18	11.93	5	0
GO: 0006508	proteolysis	14	6.50	10	6.6 × 10^−16^
GO: 0007596	blood coagulation	12	5.24	11	1.3 × 10^−10^
GO: 0022617	extracellular matrix disassembly	11	19.69	2	0
GO: 0001525	angiogenesis	10	9.05	8	0
GO: 0007275	multicellular organismal development	10	2.70	18	10^−3^
GO: 0008284	positive regulation of cell proliferation	10	5.21	12	5.1 × 10^−09^
GO: 0008285	negative regulation of cell proliferation	10	5.09	13	9.2 × 10^−09^
GO: 0044267	cellular protein metabolic process	9	3.96	14	7.9 × 10^−06^
GO: 0045944	positive regulation of transcription	9	2.43	20	5.7 × 10^−3^
GO: 0030154	cell differentiation	8	3.05	17	8.6 × 10^−4^
GO: 0030168	platelet activation	8	7.60	9	1.2 × 10^−11^
GO: 0030335	positive regulation of cell migration	8	12.46	4	0
GO: 0043066	negative regulation of apoptotic process	8	3.42	15	2.1 × 10^−4^
GO: 0001666	response to hypoxia	8	9.46	7	7.1 × 10^−15^
GO: 0007155	cell adhesion	8	3.14	16	6.3 × 10^−4^
GO: 0030574	collagen catabolic process	7	20.78	1	0
GO: 0001501	skeletal system development	7	10.08	6	3.7 × 10^−14^
GO: 0002576	platelet degranulation	7	14.26	3	0
GO: 0000122	negative regulation of transcription	7	2.63	19	7.6 × 10^−3^

^1^ Top 20 of the biological processes over-represented among the 58 dysregulated genes. ^2^ Number of genes (out of the 58 tested) involved in or belonging to the biological process stated. ^3^ The degree of enrichment (fold difference) of the relevant biological process among the subset of 58 genes, compared to the expected level. ^4^ Out of the 20 biological processed shown.

**Table 4:** GEO cellular components over-represented among the 58 newly identified markers significantly dysregulated in breast cancer.

GEO Gene Ontology Cellular Components ^1^		Counts Observed ^2^	Enrichment		*p* Value
			Fold Difference over the Expected ^3^	Rank	
GO: 0005576	extracellular region	55	8.90	8	0
GO: 0005615	extracellular space	36	8.41	9	0
GO: 0070062	extracellular vesicular exosome	26	2.56	11	4.1 × 10^−07^
GO: 0005578	proteinaceous extracellular matrix	22	19.92	3	0
GO: 0031012	extracellular matrix	20	20.99	2	0
GO: 0005604	basement membrane	8	17.21	6	0
GO: 0009986	cell surface	8	3.71	10	6.4 × 10^−05^
GO: 0005796	Golgi lumen	7	18.75	4	0
GO: 0031093	platelet α granule lumen	6	22.31	1	0
GO: 0043202	lysosomal lumen	6	17.28	5	0
GO: 0005788	endoplasmic reticulum lumen	6	8.98	7	6.6 × 10^−11^
GO: 0005789	endoplasmic reticulum membrane	6	2.23	12	4.3 × 10^−2^

^1^ All cellular components over-represented among the 58 significantly dysregulated genes (*p* < 0.05). ^2^ Genes (out of 58) categorized as encoding or associated with the cellular components stated. ^3^ The degree of enrichment (fold difference) of the relevant biological process among the subset of 58 genes, compared to the expected level.

## Data Availability

All data are showin in Supplementary Materials, uploaded with this paper.

## Supplementary Materials

The following supporting information can be downloaded at: https://www.mdpi.com/article/10.3390/genes13091538/s1, Table S1: Thirteen markers of luminal breast cancer; Table S2: 2 markers of Her2-positive breast cancer; Table S3: 8 markers of basal breast cancer.; Table S4: Predicted extracellularly targeted genes/proteins of diagnostic significance for the detection and stratification of breast cancer. Table S5: Disease association or examples of known diagnostic uses for the predicted marker genes/proteins.
