# Supplementary material for: Rational Approach to Finding Genes Encoding Molecular Biomarkers: Focus on Breast Cancer
Source: Genes (Basel). 2022 Aug 26;13(9):1538. doi: 10.3390/genes13091538 (PMC9498645; doi:10.3390/genes13091538)
Supplement: Supplementary file 1 [file genes-13-01538-s001.zip › genes-1855831-supplementary.pdf]

**Table S1.** Thirteen markers of luminal breast cancer.

| Marker | Protein function and typical diagnostic uses |
|---|---|
| ESR1 | Estrogen Receptor 1: A nuclear hormone receptor acting as a transcription factor, and influencing cell proliferation and differentiation. ESR1 has a typical steroid receptor structure and can bind to DNA. ESR1 increases the expression of p53, a common tumour protein. |
| ESR2 | Estrogen Receptor 2: A nuclear receptor transcription factor. ESR2 expression is a prognostic marker in breast cancer, associated with positive outcome. ESR2 is highly expressed in normal breast tissue, but expression is decreased in cancerous tissue. It is significantly different from ESR1. |
| PGR | Progesterone Receptor: A steroid receptor. It is involved in cell proliferation and differentiation, as well as mediating the effect of progesterone in pregnancy. PGR is a drug-target gene and involved in the classification as luminal-subtype cancers. |
| CCND1 | Cyclin D1: A member of the cyclin family. CCND1 is important in the regulation of the cell cycle. It can be used in the identification of lobular carcinoma in situ, which is likely to become invasive. |
| FOXA1 | Forkhead Box A1: A hepatocyte nuclear transcription factor involved in gene expression in embryonic and differentiated tissue. FOXA1 is a transcription factor and has been shown to be coexpressed with ESR1. |
| GATA3 | GATA Binding Protein 3: A transcription factor regulating T cell development. GATA3 is an important oncogene and promising drug target for breast cancer. GATA3 also is a transcription factor and coexpressed with ESR1. |
| KRT18 | Keratin 18: A member of the intermediate filament family and dimerization partner of KRT8. KRT18 has been shown to be associated with a reduced invasiveness. |
| KRT8 | Keratin 8: A member of the type II keratin family and dimerization partner of KRT18. KRT8 is expressed in the differentiation compartment and linked to good prognosis. |
| LAPTM4B | Lysosomal Protein Transmembrane 4 Beta: A protein linked to hepatocellular carcinoma. LAPTM4B is upregulated in a variety of tumours, including lung, colon and luminal breast cancer. |
| SLC39A6 | Solute Carrier Family 39 Member 6: A transmembrane transporter of zinc. SLC39A6 is associated with a positive outcome and in combination with ESR1 is predictive of relapse free survival. |
| SQLE | Squalene Epoxidase: An enzyme involved in sterol biosynthesis. SQLE is overexpressed in aggressive breast cancer and an independent prognostic marker of negative outcome. |
| TFF3 | Trefoil Factor 3: A stable secretory protein. TFF3 is a biomarker for metastatic breast cancer response to endocrine therapy. |
| XBP1 | X-Box Binding Protein 1: A transcription factor active during endoplasmic reticulum stress. XBP1 has been shown to be co-expressed with ESR1 and be involved in its actions. |

**Table S2.** Two markers of Her2-positive breast cancer.

| Marker | Protein function and typical diagnostic uses |
|---|---|
| ERBB2 | Erb-B2 Receptor Tyrosine Kinase 2: An epidermal growth factor receptor, which requires a coreceptor. ERBB2 is a drug-target gene and essential for classification as HER2-subtype. |
| GRB7 | Growth Factor Receptor Bound Protein 7: An adapter protein interacting with receptor tyrosine kinases and signalling molecules. GRB7 has been shown to correlate with transcript levels of ERBB2 in inflammatory breast cancer. |

**Table S3.** Eight markers of basal breast cancer.

| Marker | Protein function and typical diagnostic uses |
|---|---|
| KRT5 | Keratin 5: A type II keratin family member with structural and scaffolding properties. KRT5 is expressed in proliferation compartment. KRT5 is associated with triple-negative breast cancer and good prognosis. |
| CDH3 | Cadherin 3: A cell adhesion molecule dependent on calcium. CDH3 is overexpressed in breast carcinomas and associated with high proliferation and poor survival. |
| ID4 | Inhibitor of DNA Binding 4, HLH Protein: A transcription factor, which requires another bound protein and is involved in tumour suppression. ID4 inhibits BRCA1 function in basal subtype breast cancers. ID4 expression correlates with poor prognosis. |
| FABP7 | Fatty Acid Binding Protein 7: A cytoplasmic protein binding hydrophobic molecules and important in brain development. FABP7 expression may induce metastasis. FABP7 is involved in a variety of human cancers. |
| KRT17 | Keratin 17: A type I keratin important in skin and hair structure. KRT17 is expressed at varying sites of breast tissue, but levels are generally low. |
| TRIM29 | Tripartite Motif Containing 29: A protein forming various dimers for DNA binding. TRIM29 expression is correlated to relapse-free survival and a role as tumour suppressor has been proposed. |
| LAMC2 | Laminin Subunit Gamma 2: An extracellular matrix constituent involved in cell adhesion and migration. LAMC2 is a typical cancer invasion marker and stimulates the migration of cells via CD44. |
| ITGB4 | Integrin Subunit Beta 4: A transmembrane receptor involved in cell adhesion and signalling pathways. ITGB4 affects the expression of SPARC and invasiveness of breast cancer. |

**Table S4.** Predicted extracellularly targeted genes/proteins of diagnostic significance for the detection and stratification of breast cancer.

| **Gene** | **UniProt ID** | **Protein Name** | **HG-U133A*** | **B** | **L** | **H** |
|---|---|---|---|---|---|---|
| *ADAMTS1* | Q9UHI8 | A disintegrin and metalloproteinase with thrombospondin motifs 1 | 222162_s_at | X | | |
| *ADAMTS17* | Q8TE56 | A disintegrin and metalloproteinase with thrombospondin motifs 17 | n/a** | | | |
| *ADM* | P35318 | ADM | 202912_at | X | | |
| *AGR2* | O95994 | Anterior gradient protein 2 homolog | 209173_at | | X | |
| *ALPI* | P09923 | Intestinal-type alkaline phosphatase | 207140_at | X | | |
| *ANG* | P03950 | Angiogenin | 205141_at | | X | |
| *APOA5* | Q6Q788 | Apolipoprotein A-V | n/a** | | | |
| *APOB* | P04114 | Apolipoprotein B-100 | 205108_s_at | | X | |
| *APOC3* | P02656 | Apolipoprotein C-III | 205820_s_at | | X | |
| *AVP* | P01185 | Vasopressin-neurophysin 2-copeptin | 207848_at | | X | |
| *BGN* | P21810 | Biglycan | 201262_s_at | X | | |
| *BMP2* | P12643 | Bone morphogenetic protein 2 | 205289_at | X | | |
| *BMP4* | P12644 | Bone morphogenetic protein 4 | 211518_s_at | X | | |
| *C1S* | P09871 | Complement C1s subcomponent | 208747_s_at | | X | |
| *CCK* | P06307 | Cholecystokinin | 205827_at | X | | |
| *CCL2* | P13500 | C-C motif chemokine 2 | 216598_s_at | X | | |
| *CCL5* | P13501 | C-C motif chemokine 5 specific protein | 204655_at | | X | |
| *CD24* | P25063 | Signal transducer CD24 | 208650_s_at | | X | |
| *CD59* | P13987 | CD59 glycoprotein | 212463_at | | X | |
| *CDH13* | P55290 | Cadherin-13 | 204726_at | X | | |
| *CEMIP* | Q8WUJ3 | Cell migration-inducing and hyaluronan-binding [illegible] | 212942_s_at | X | | |
| *CGA* | P01215 | Glycoprotein hormones alpha chain | 204637_at | | X | |
| *CHRDL1* | Q9BU40 | Chordin-like protein 1 | 209763_at | X | | |
| *COL1A1* | P02452 | Collagen alpha-1(I) chain | 202312_s_at | X | X | |
| *COL1A2* | P08123 | Collagen alpha-2(I) chain | 202404_s_at | X | X | |
| *COL2A1* | P02458 | Collagen alpha-1(II) chain | 213492_at | X | | |
| *COL4A1* | P02462 | Collagen alpha-1(IV) chain | 211981_at | X | X | |
| *COL4A2* | P08572 | Collagen alpha-2(IV) chain | 211964_at | X | X | |
| *COL4A3* | Q01955 | Collagen alpha-3(IV) chain | 214641_at | X | | |
| *COL5A1* | P20908 | Collagen alpha-1(V) chain | 212488_at | X | | |
| *CRH* | P06850 | Corticoliberin | 205630_at | X | | |
| *CRP* | P02741 | C-reactive protein | 205753_at | X | | |
| *CSF2* | P04141 | Granulocyte-macrophage colony-stimulating [illegible] | 210228_at | X | | |
| *CSF3* | P09919 | Granulocyte colony-stimulating factor | 207442_at | X | | |
| *CTGF* | P29279 | Connective tissue growth factor | 209101_at | X | | |

| | | | | | | |
|---|---|---|---|---|---|---|
| *CTSD* | P07339 | Cathepsin D | 200766_at | | X | |
| *CXCL1* | P09341 | Growth-regulated alpha protein | 204470_at | X | | |
| *CXCL10* | P02778 | C-X-C motif chemokine 10 | 204533_at | X | | |
| *CXCL12* | P48061 | Stromal cell-derived factor 1 | 203666_at | X | X | |
| *CXCL14* | O95715 | C-X-C motif chemokine 14 | 218002_s_at | X | | |
| *CXCL5* | P42830 | C-X-C motif chemokine 5 | 207852_at | X | | |
| *CXCL8* | P10145 | Interleukin-8 | 211506_s_at | X | | |
| *CYR61* | O00622 | Protein CYR61 | 201289_at | X | | |
| *DCN* | P07585 | Decorin | 209335_at | X | | |
| *DEFA4* | P12838 | Neutrophil defensin 4 | 207269_at | | X | |
| *DKK1* | O94907 | Dickkopf-related protein 1 | 204602_at | X | | |
| *EDEM2* | Q9BV94 | ER degradation-enhancing alpha-mannosidase-like protein 2 | 218282_at | | X | |
| *EDN1* | P05305 | Endothelin-1 | 218995_s_at | X | | |
| *ELN* | P15502 | Elastin | 212670_at | X | | |
| *EPO* | P01588 | Erythropoietin | 207257_at | X | X | X |
| *ERP29* | P30040 | Endoplasmic reticulum resident protein 29 | 201216_at | | X | |
| *F7* | P08709 | Coagulation factor VII | 207300_s_at | X | | |
| *F9* | P00740 | Coagulation factor IX | 207218_at | X | | |
| *FBLN1* | P23142 | Fibulin-1 | 201787_at | X | | |
| *FGF21* | Q9NSA1 | Fibroblast growth factor 21 | 221433_at | X | | |
| *FGFBP1* | Q14512 | Fibroblast growth factor-binding protein 1 | 205014_at | X | | |
| *FIGF* | O43915 | Vascular endothelial growth factor D | 206742_at | X | | |
| *FN1* | P02751 | Fibronectin | 214702_at | X | | X |
| *FST* | P19883 | Follistatin | 207345_at | X | | |
| *GCG* | P01275 | Glucagon | 206422_at | | X | |
| *GH2* | P01242 | Growth hormone variant | 211508_s_at | X | | |
| *GNAS* | O95467 | Neuroendocrine secretory protein 55 | 214157_at | X | | |
| *GNRH1* | P01148 | Progonadoliberin-1 | 207987_s_at | X | | |
| *GNRH2* | O43555 | Progonadoliberin-2 | 208519_x_at | | | X |
| *HPSE* | Q9Y251 | Heparanase | 219403_s_at | X | | |
| *HSPA5* | P11021 | 78 kDa glucose-regulated protein | 211936_at | | X | |
| *IFNG* | P01579 | Interferon gamma | 210354_at | | X | |
| *IGF1* | P05019 | Insulin-like growth factor I | 209540_at | X | | |
| *IGFBP3* | P17936 | Insulin-like growth factor-binding protein 3 | 210095_s_at | X | | |
| *IGFBP4* | P22692 | Insulin-like growth factor-binding protein 4 | 201508_at | X | | |
| *IGFBP5* | P24593 | Insulin-like growth factor-binding protein 5 | 203425_s_at | X | | X |
| *IL11* | P20809 | Interleukin-11 | 206924_at | X | | |
| *IL17D* | Q8TAD2 | Interleukin-17D | n/a** | | | |

| | | | | | | |
|---|---|---|---|---|---|---|
| *IL18BP* | O95998 | Interleukin-18-binding protein | 219323_s_at | | X | |
| *IL21* | Q9HBE4 | Interleukin-21 | 221271_at | X | | |
| *IL5* | P05113 | Interleukin-5 | 207952_at | | X | |
| *IL6* | P05231 | Interleukin-6 | 205207_at | X | | |
| *INHBA* | P08476 | Inhibin beta A chain | 204926_at | X | | |
| *KISS1* | Q15726 | Metastasis-suppressor KiSS-1 | 205563_at | X | | |
| *KLK1* | P06870 | Kallikrein-1 | 216699_s_at | | X | |
| *KLK3* | P07288 | Prostate-specific antigen | 204582_s_at | X | X | X |
| *LAMC2* | Q13753 | Laminin subunit gamma-2 | 202267_at | X | | |
| *LCN2* | P80188 | Neutrophil gelatinase-associated lipocalin | 212531_at | X | | |
| *LEP* | P41159 | Leptin | 207092_at | X | | |
| *LOX* | P28300 | Protein-lysine 6-oxidase | 204298_s_at | X | | |
| *LOXL2* | Q9Y4K0 | Lysyl oxidase homolog 2 | 202998_s_at | | X | |
| *LPL* | P06858 | Lipoprotein lipase | 203549_s_at | X | | |
| *LRPAP1* | P30533 | Alpha-2-macroglobulin receptor-associated [illegible] | 201186_at | | X | |
| *LTBP1* | Q14766 | Latent-transforming growth factor beta-binding [illegible] | 202729_s_at | X | | |
| *LTBP3* | Q9NS15 | Latent-transforming growth factor beta-binding [illegible] | 219922_s_at | X | | |
| *LTF* | P02788 | Lactotransferrin | 202018_s_at | X | | |
| *LUM* | P51884 | Lumican | 201744_s_at | X | | |
| *MFGE8* | Q08431 | Lactadherin | 210605_s_at | X | | |
| *MMP1* | P03956 | Interstitial collagenase | 204475_at | X | | |
| *MMP10* | P09238 | Stromelysin-2 | 205680_at | X | | |
| *MMP11* | P24347 | Stromelysin-3 | 203878_s_at | X | | |
| *MMP12* | P39900 | Macrophage metalloelastase | 204580_at | X | | |
| *MMP13* | P45452 | Collagenase 3 | 205959_at | X | | |
| *MMP2* | P08253 | 72 kDa type IV collagenase | 201069_at | X | | |
| *MMP20* | O60882 | Matrix metalloproteinase-20 | 207599_at | X | | |
| *MMP28* | Q9H239 | Matrix metalloproteinase-28 | 219909_at | X | | |
| *MMP3* | P08254 | Stromelysin-1 | 205828_at | X | | |
| *MMP7* | P09237 | Matrilysin | 204259_at | X | | |
| *MMP9* | P14780 | Matrix metalloproteinase-9 | 203936_s_at | X | | |
| *NID1* | P14543 | Nidogen-1 | 202007_at | X | | |
| *NPPB* | P16860 | Natriuretic peptides B | 206801_at | | X | |
| *NTS* | P30990 | Neurotensin/neuromedin N | 206291_at | X | | |
| *OGN* | P20774 | Mimecan | 218730_s_at | X | | |
| *PDGFA* | P04085 | Platelet-derived growth factor subunit A | 205463_s_at | X | X | |
| *PDGFB* | P01127 | Platelet-derived growth factor subunit B | 216055_at | X | X | |
| *PDGFD* | Q9GZP0 | Platelet-derived growth factor D | 219304_s_at | X | | |
| *PENK* | P01210 | Proenkephalin-A | 213791_at | X | | |

| | | | | | | |
|---|---|---|---|---|---|---|
| *PLAU* | P00749 | Urokinase-type plasminogen activator | 205479_s_at | X | | |
| *PLAUR* | Q03405 | Urokinase plasminogen activator surface | 214866_at | X | | |
| *POMC* | P01189 | Pro-opiomelanocortin | 205720_at | X | | |
| *PROS1* | P07225 | Vitamin K-dependent protein S | 207808_s_at | X | | |
| *PTGDS* | P41222 | Prostaglandin-H2 D-isomerase | 211663_x_at | X | | |
| *PTGS2* | P35354 | Prostaglandin G/H synthase 2 | 204748_at | X | | |
| *PTHLH* | P12272 | Parathyroid hormone-related protein | 210355_at | | X | |
| *PTN* | P21246 | Pleiotrophin | 208408_at | X | | |
| *RBP3* | P10745 | Retinol-binding protein 3 | 210318_at | X | | |
| *RELN* | P78509 | Reelin | 205923_at | X | | |
| *RGMA* | Q96B86 | Repulsive guidance molecule A | n/a** | | | |
| *RLN1* | P04808 | Prorelaxin H1 | 211753_s_at | | X | |
| *RLN2* | P04090 | Prorelaxin H2 | 214519_s_at | | X | |
| *SERPINA1* | P01009 | Alpha-1-antitrypsin | 211428_at | | X | |
| *SERPINB2* | P05120 | Plasminogen activator inhibitor 2 | 204614_at | X | | |
| *SERPINE1* | P05121 | Plasminogen activator inhibitor 1 | 202628_s_at | X | X | |
| *SLPI* | P03973 | Antileukoproteinase | 203021_at | | X | |
| *SOD3* | P08294 | Extracellular superoxide dismutase | 205236_x_at | X | | |
| *SPARC* | P09486 | SPARC | 212667_at | X | | |
| *SPARCL1* | Q14515 | SPARC-like protein 1 | 200795_at | | X | |
| *SULF2* | Q8IWU5 | Extracellular sulfatase Sulf-2 | n/a** | | | |
| *TF* | P02787 | Serotransferrin | 214064_at | X | | |
| *TFF1* | P04155 | Trefoil factor 1 | 205009_at | | X | X |
| *TFF2* | Q03403 | Trefoil factor 2 | 214476_at | X | X | |
| *TFF3* | Q07654 | Trefoil factor 3 | 204623_at | | X | |
| *TGFB1* | P01137 | Transforming growth factor beta-1 | 203084_at | X | X | |
| *TGFB2* | P61812 | Transforming growth factor beta-2 | 220406_at | X | | |
| *TGFBI* | Q15582 | Transforming growth factor-beta-induced protein | 201506_at | X | | |
| *TIMP1* | P01033 | Metalloproteinase inhibitor 1 | 201666_at | X | | |
| *TIMP3* | P35625 | Metalloproteinase inhibitor 3 | 201150_s_at | | X | |
| *TNC* | P24821 | Tenascin | 201645_at | X | | |
| *TNFAIP6* | P98066 | Tumor necrosis factor-inducible gene 6 protein | 206026_s_at | X | | |
| *TNXB* | P22105 | Tenascin-X | 216654_at | X | | |
| *TRH* | P20396 | Pro-thyrotropin-releasing hormone | 206622_at | | X | |
| *VEGFA* | P15692 | Vascular endothelial growth factor A | 210512_s_at | X | X | X |
| *WNT5A* | P41221 | Protein Wnt-5a | 213425_at | X | | |
| *WNT6* | Q9Y6F9 | Protein Wnt-6 | 221608_at | X | | |
| *WNT9A* | O14904 | Protein Wnt-9a | n/a** | | | |

* Affymetrix Human Genome U133A Array
** Not found in Affymetrix Human Genome U133A Array

**Table S5.** Disease association or examples of known diagnostic uses for the predicted marker genes/proteins

| Gene | UniProt | Disease association or examples of known or proposed diagnostic uses | Gene/protein information, publications |
|---|---|---|---|
| *ADAMTS1* | Q9UHI8 | Potentially acute aortic dissection, inflammation, cancer cachexia, possibly breast/liver carcinoma, NSCLC. Play crucial role in colorectal cancer etiopathogenesis. May act as t*umor gene* in human breast carcinoma | https://www.uniprot.org/uniprotkb/Q9UHI8 |
| *ADAMTS-1* | Q8TE56 | Potentially diseases of the eye. Weill-Marchesani syndrome. May support breast cancer cell growth and survival. Inverse correlation between higher Adamts17 expression and patients' survival. | https://www.uniprot.org/uniprotkb/Q8TE56 |
| *ADM* | P35318 | Early ectopic pregnancy, steroid secretion deficiency, sepsis, biomarker for acute intracerebral hemorrhage. Promotes tumor growth. Expression correlates with tumor and lymph node lymphangiogenesis. Predictor of 10-year all-cause mortality | https://www.uniprot.org/uniprotkb/P35318 |
| *AGR2* | O95994 | Proto-oncogene, pituitary adenomas, overexpressed in ER+ breast tumors, other cancers; promotes tumor growth, cell migration, and cellular transformation | https://www.uniprot.org/uniprotkb/O95994 |
| *ALPI* | P09923 | Liver, bone, parathyroid and intestinal diseases, linked to tumor progression in different cancers | https://www.uniprot.org/uniprotkb/P09923 |
| *ANG* | P03950 | Detection of prostate cancer, associated with amyotrophic lateral sclerosis | https://www.uniprot.org/uniprotkb/P03950 |
| *APOA5* | Q6Q788 | Hyperlipoproteinemia, coronary artery diseases. Immunomodulatory effect on the tumor microenvironment. Copy number loss prevalent in luminal breast tumors | https://www.uniprot.org/uniprotkb/Q6Q788 |
| *APOB* | P04114 | Hypercholesterolemia, hyperlipidemia, acanthocytosis with hypobetalipoproteinemia, hypertriglyceridemia. Used in test for risk of cardiovascular disease. Low expression or hypermethylation might predict survival in low-grade glioma patients. Polymorphisms increase the risk of breast cancer | https://www.uniprot.org/uniprotkb/P04114 |
| *APOC3* | P02656 | Amyloidosis, cardiovascular diseases. Potential use for differentiation of small cell lung cancer | https://www.uniprot.org/uniprotkb/P02656 |
| *AVP* | P01185 | Hypertensive diseases. Diagnosis of diabetes insipidus and CVD | https://www.uniprot.org/uniprotkb/P01185 |
| *BGN* | P21810 | Potentially fibrosis in hepatitis B, Spondyloepimetaphyseal Dysplasia, Meester-Loeys syndrome, aortic aneurysm/dissection. Upregulated in colorectal cancers, endometrial cancer. Potential diagnostic and prognostic biomarker in multiple human cancers. | https://www.uniprot.org/uniprotkb/P21810 |
| *BMP2* | P12643 | Congenital diseases, mammary and digestive system neoplasms. RhBMP2 for treatment of open tibial fractures | https://www.uniprot.org/uniprotkb/P12643 |
| *BMP4* | P12644 | Various bone diseases, congenital diseases, developmental delay. Linked to reduced proliferation and increased recurrence in breast cancer | https://www.uniprot.org/uniprotkb/P12644 |
| *C1S* | P09871 | C1s deficiency, SLE and glomerulonephritis, Ehlers-Danlos syndrome, lupus. Promote growth of cutaneous squamous cell carcinoma. Prognostic Biomarkers in Renal Cancer | https://www.uniprot.org/uniprotkb/P09871 |
| *CCK* | P06307 | Tumors and cholecystokininoma | https://www.uniprot.org/uniprotkb/P06307 |
| *CCL2* | P13500 | Malignant pleural mesothelomia | https://www.uniprot.org/uniprotkb/P13500 |
| *CCL5* | P13501 | Hepatocellular carcinoma, asthma, hyperoxaluria | https://www.uniprot.org/uniprotkb/P13501 |
| *CD24* | P25063 | NSC lung cancer, pancreatic neoplasms | https://www.uniprot.org/uniprotkb/P25063 |
| *CD59* | P13987 | Chronic Hemolysis, FCM for Nocturnal Hemoglobinuria. Promotes tumor growth and predicts poor prognosis in breast cancer | https://www.uniprot.org/uniprotkb/P13987 |
| *CDH13* | P55290 | Lung neoplasms, NSCLC | https://www.uniprot.org/uniprotkb/P55290 |

| | | | |
|---|---|---|---|
| *CEMIP* | Q8WUJ3 | Potentially colon cancer prognosis | https://www.uniprot.org/uniprotkb/Q8WUJ3 |
| *CGA* | P01215 | Associated with adenocarcinoma | https://www.uniprot.org/uniprotkb/P01215 |
| *CHRDL1* | Q9BU40 | Mutation in megalocornea, congenital keratoglobus. Associated with malignant biological behaviors in multiple cancers. | https://www.uniprot.org/uniprotkb/Q9BU40 |
| *COL1A1* | P02452 | Lobstein disease, osteogenesis imperfecta, Ehlers-Danlos syndrome, aortic aneurysm/dissection. Hepatocellular Carcinogenesis and Metastasis. Promotes Metastasis of Breast Cancer. | https://www.uniprot.org/uniprotkb/P02452 |
| *COL1A2* | P08123 | Osteogenesis imperfecta, osteoporosis, Ehlers-Danlos syndrome. Inhibitory effects on colorectal cancer. Expresison correlates with proliferation and metastasis of esophageal cancer. Prognostic biomarker in lung cancer. Reported to be involved in multiple other cancers (skin, bone, pancreatic, lung, liver, prostate, stomach, etc) | https://www.uniprot.org/uniprotkb/P08123 |
| *COL2A1* | P02458 | Achondrogenesis, spondyloepiphyseal dysplasia, stickler syndrome, multiple congenital, hereditary and neonatal diseases. Predictor of tumor recurrence in high-grade ovarian cancer. Biomarker of Melanoma Tumor Repopulating Cells | https://www.uniprot.org/uniprotkb/P02458 |
| *COL4A1* | P02462 | Thyroid and liver cancer, multiple congenital, hereditary, neonatal and nervous system diseases | https://www.uniprot.org/uniprotkb/P02462 |
| *COL4A2* | P08572 | Colon cancer, porencephaly | https://www.uniprot.org/uniprotkb/P08572 |
| *COL4A3* | Q01955 | Autosomal recessive Alport syndrome. Nasopharyngeal carcinoma. Potential prognostic biomarker for gastric cancer | https://www.uniprot.org/uniprotkb/Q01955 |
| *COL5A1* | P20908 | Ehlers-Danlos syndrome. Prognostic and Immunological Biomarker for Multiple Human Tumors | https://www.uniprot.org/uniprotkb/P20908 |
| *CRH* | P06850 | Mental disorders, Cushing's disease. CRH signaling affected in multiple cancers | https://www.uniprot.org/uniprotkb/P06850 |
| *CRP* | P02741 | Inflammation, obesity, cardiovascular diseases. Prognostic biomarker in most adult solid tumors | https://www.uniprot.org/uniprotkb/P02741 |
| *CSF2* | P04141 | Linked to multiple neoplasms. Prognosis in tuberculosis | https://www.uniprot.org/uniprotkb/P04141 |
| *CSF3* | P09919 | Linked to multiple neoplasms. Prognosis in tuberculosis | https://www.uniprot.org/uniprotkb/P09919 |
| *CTGF* | P29279 | Cardiovascular and hematological diseases, colorectal cancer. Therapeutic target for cancer, fibrosis and other related disorders in a variety of tissues | https://www.uniprot.org/uniprotkb/P29279 |
| *CTSD* | P07339 | Nasopharyngeal carcinoma, liver carcinoma, neuronal ceroid lipofuscinosis | https://www.uniprot.org/uniprotkb/P07339 |
| *CXCL1* | P09341 | Ovarian carcinoma differentiation | https://www.uniprot.org/uniprotkb/P09341 |
| *CXCL10* | P02778 | Potentially neurosyphilis. Responsible for recruitment of tumor antigen specific CD8+ T cells in the tumor microenvironment. Potential biomarker associated with immune infiltration. | https://www.uniprot.org/uniprotkb/P02778 |
| *CXCL12* | P48061 | Linked to multiple neoplasms. Ischemic stroke severity | https://www.uniprot.org/uniprotkb/P48061 |
| *CXCL14* | O95715 | Potentially colorectal carcinoma, lung/liver neoplasms | https://www.uniprot.org/uniprotkb/O95715 |
| *CXCL5* | P42830 | Potentially bladder cancer | https://www.uniprot.org/uniprotkb/P42830 |
| *CXCL8* | P10145 | Potentially neurosyphilis. Promotes tumor survival, facilitates tumor cell proliferation and epithelial-to-mesenchymal transition, pro-angiogenesis, inhibits anti-tumor immunity | https://www.uniprot.org/uniprotkb/P10145 |
| *CYR61* | O00622 | Acute renal failure. Associated with prognosis in colorectal and breast cancers. Potential biomarker of tumor inflammatory response | https://www.uniprot.org/uniprotkb/O00622 |
| *DCN* | P07585 | Corneal dystrophy, congenital stromal. Potentially colon cancer | https://www.uniprot.org/uniprotkb/P07585 |
| *DEFA4* | P12838 | Mammary neoplasms, salivary gland tumors | https://www.uniprot.org/uniprotkb/P12838 |

| | | | |
|---|---|---|---|
| *DKK1* | O94907 | Cancers, such as liver cancer | https://www.uniprot.org/uniprotkb/O94907 |
| *EDEM2* | Q9BV94 | Inflammation | https://www.uniprot.org/uniprotkb/Q9BV94 |
| *EDN1* | P05305 | Hypertension, auriculo-condylar syndrome. stimulates the proliferation of many malignant cells | https://www.uniprot.org/uniprotkb/P05305 |
| *ELN* | P15502 | Ventilator associated pneumonia, aortic diseases, respiratory tract diseases. Colorectal cancer, leukemias | https://www.uniprot.org/uniprotkb/P15502 |
| *EPO* | P01588 | Anemia, familial erythrocytosis | https://www.uniprot.org/uniprotkb/P01588 |
| *ERP29* | P30040 | Colorectal cancer, adenocarcinoma of lung | https://www.uniprot.org/uniprotkb/P30040 |
| *F7* | P08709 | Hemophilia recovery and bleeding risk. Factor VII Deficiency. associated with better relapse-free survival in breast cancer | https://www.uniprot.org/uniprotkb/P08709 |
| *F9* | P00740 | Hemophilia, thrombophilia. Overexpressed in teratoma cell lines. | https://www.uniprot.org/uniprotkb/P00740 |
| *FBLN1* | P23142 | Prostate cancer, malignant neoplasm of prostate, synpolydactyly | https://www.uniprot.org/uniprotkb/P23142 |
| *FGF21* | Q9NSA1 | Potentially renal cancer | https://www.uniprot.org/uniprotkb/Q9NSA1 |
| *FGFBP1* | Q14512 | Metastasis in gallbladder cancer | https://www.uniprot.org/uniprotkb/Q14512 |
| *FIGF* | O43915 | Endothelial cell function assays | https://www.uniprot.org/uniprotkb/O43915 |
| *FN1* | P02751 | Spondylometaphyseal dysplasia, glomerulopathy with fibronectin deposits, multiple carcinomas, glioblastoma. | https://www.uniprot.org/uniprotkb/P02751 |
| *FST* | P19883 | Ovarian endometrioma, breast and liver neoplasms and carcinomas | https://www.uniprot.org/uniprotkb/P19883 |
| *GCG* | P01275 | Pancreas tumor | https://www.uniprot.org/uniprotkb/P01275 |
| *GH2* | P01242 | Diabetic pregnancy. Overexpressed in basal cell carcinoma, prostate carcinoma, chronic adult T-cell leukemia/lymphoma, bladder urothelial carcinoma, colon mucinous adenocarcinoma, ovarian carcinomas | https://www.uniprot.org/uniprotkb/P01242 |
| *GNAS* | O95467 | Pseudohypoparathyroidism. Mutation in thyroid cancer | https://www.uniprot.org/uniprotkb/O95467 |
| *GNRH1* | P01148 | Familial hypogonadotropic eunuchoidism, hypogonadotropic hypogonadism. Mutation in CHH | https://www.uniprot.org/uniprotkb/P01148 |
| *GNRH2* | O43555 | Potentially bone neoplasms | https://www.uniprot.org/uniprotkb/O43555 |
| *HPSE* | Q9Y251 | Tumor metastasis and invasion, liver and breast carcinomas, melanoma | https://www.uniprot.org/uniprotkb/Q9Y251 |
| *HSPA5* | P11021 | Hepatocellular carcinoma | https://www.uniprot.org/uniprotkb/P11021 |
| *IFNG* | P01579 | Tuberculosis, aplastic anemia. Induces apoptosis or suppresses cancer cells, affects cancer immunotherapy. May contribute to tumor metastasis, immune escape. | https://www.uniprot.org/uniprotkb/P01579 |
| *IGF1* | P05019 | GH deficiency and hypopituitarism, prostatic and breasts neoplasms | https://www.uniprot.org/uniprotkb/P05019 |
| *IGFBP3* | P17936 | GH deficiency, colorectal carcinoma, | https://www.uniprot.org/uniprotkb/P17936 |
| *IGFBP4* | P22692 | Hormonal therapy in BC prediction | https://www.uniprot.org/uniprotkb/P22692 |
| *IGFBP5* | P24593 | Hormonal therapy in BC prediction, mammary neoplasms | https://www.uniprot.org/uniprotkb/P24593 |
| *IL11* | P20809 | Lung adenocarcinoma and BALF | https://www.uniprot.org/uniprotkb/P20809 |
| *IL17D* | Q8TAD2 | Inflammation | https://www.uniprot.org/uniprotkb/Q8TAD2 |
| *IL18BP* | O95998 | CHD development | https://www.uniprot.org/uniprotkb/O95998 |
| *IL21* | Q9HBE4 | Inflammation and immune responses, immunodeficiency | https://www.uniprot.org/uniprotkb/Q9HBE4 |
| *IL5* | P05113 | Severity of sepsis, asthma, eosinophilia | https://www.uniprot.org/uniprotkb/P05113 |
| *IL6* | P05231 | Differentiation of pancreatic cancer, multiple other pathological conditions and cardiovascular diseases | https://www.uniprot.org/uniprotkb/P05231 |
| *INHBA* | P08476 | Prognosis in urothelial carcinoma | https://www.uniprot.org/uniprotkb/P08476 |
| *KISS1* | Q15726 | Breast cancer metastasis, hypogonadotropic hypogonadism | https://www.uniprot.org/uniprotkb/Q15726 |
| *KLK1* | P06870 | Carcinogenesis | https://www.uniprot.org/uniprotkb/P06870 |

| | | | |
|---|---|---|---|
| *KLK3* | P07288 | Prostatic neoplasms. Prostate cancer screening | https://www.uniprot.org/uniprotkb/P07288 |
| *LAMC2* | Q13753 | Herlitz disease, epidermolysis | https://www.uniprot.org/uniprotkb/Q13753 |
| *LCN2* | P80188 | Potentially cholangiocarcinoma, kidney diseases | https://www.uniprot.org/uniprotkb/P80188 |
| *LEP* | P41159 | Leptin deficiency/disfunction, hyperinsulinism. Treatment of hypoleptinemic states | https://www.uniprot.org/uniprotkb/P41159 |
| *LOX* | P28300 | Atherosclerosis, arthritis, aortic aneurysms | https://www.uniprot.org/uniprotkb/P28300 |
| *LOXL2* | Q9Y4K0 | Breast carcinoma, tumor progression | https://www.uniprot.org/uniprotkb/Q9Y4K0 |
| *LPL* | P06858 | Chronic lymphocytic leukemia, hyperlipidemia, hyperlipoproteinemia | https://www.uniprot.org/uniprotkb/P06858 |
| *LRPAP1* | P30533 | Brain diseases and atherosclerosis | https://www.uniprot.org/uniprotkb/P30533 |
| *LTBP1* | Q14766 | Breast cancer metastasis | https://www.uniprot.org/uniprotkb/Q14766 |
| *LTBP3* | Q9NS15 | Verloes Bourguignon syndrome, amelogenesis imperfecta. Prognosis in hepatocellular carcinomas | https://www.uniprot.org/uniprotkb/Q9NS15 |
| *LTF* | P02788 | Gastrointestinal inflammation | https://www.uniprot.org/uniprotkb/P02788 |
| *LUM* | P51884 | Liver diseases, breast cancer | https://www.uniprot.org/uniprotkb/P51884 |
| *MFGE8* | Q08431 | Breast carcinomas and angiogenesis | https://www.uniprot.org/uniprotkb/Q08431 |
| *MMP1* | P03956 | Chronic obstructive airway disease, Hallopeau-Siemens disease, mammary neoplasms, oral cancer | https://www.uniprot.org/uniprotkb/P03956 |
| *MMP10* | P09238 | Malignancy of pleural effusion, linked to multiple neoplasms | https://www.uniprot.org/uniprotkb/P09238 |
| *MMP11* | P24347 | Colorectal cancer, non-small cell lung carcinoma | https://www.uniprot.org/uniprotkb/P24347 |
| *MMP12* | P39900 | Polymorphism in COPD, pulmonary emphysema | https://www.uniprot.org/uniprotkb/P39900 |
| *MMP13* | P45452 | Prostate cancer, metaphyseal chondrodysplasia | https://www.uniprot.org/uniprotkb/P45452 |
| *MMP2* | P08253 | Colorectal cancer, linked to multiple other neoplasms, neoplasm metastasis, Torg-Winchester syndrome | https://www.uniprot.org/uniprotkb/P08253 |
| *MMP20* | O60882 | Potentially various tumors, amelogenesis imperfecta | https://www.uniprot.org/uniprotkb/O60882 |
| *MMP28* | Q9H239 | Potentially colon cancer | https://www.uniprot.org/uniprotkb/Q9H239 |
| *MMP3* | P08254 | Oral cancer, hyperalgesia | https://www.uniprot.org/uniprotkb/P08254 |
| *MMP7* | P09237 | Potentially choliangiocarcinoma, other neoplasms of digestive system | https://www.uniprot.org/uniprotkb/P09237 |
| *MMP9* | P14780 | Cardiovascular diseases, colonic neoplasms, neoplasm metastasis. Prostate cancer prognosis | https://www.uniprot.org/uniprotkb/P14780 |
| *NID1* | P14543 | Dandy-Walker Syndrome. Potentially ovarian cancer | https://www.uniprot.org/uniprotkb/P14543 |
| *NPPB* | P16860 | Heart failure, cardiovascular diseases | https://www.uniprot.org/uniprotkb/P16860 |
| *NTS* | P30990 | Various malignant tumors, schizophrenia | https://www.uniprot.org/uniprotkb/P30990 |
| *OGN* | P20774 | Heart failure, neoplasm metastasis | https://www.uniprot.org/uniprotkb/P20774 |
| *PDGFA* | P04085 | Several cancers and atherosclerosis | https://www.uniprot.org/uniprotkb/P04085 |
| *PDGFB* | P01127 | Dermatofibrosarcoma protuberans, giant cell fibroblastoma, Fahr's syndrome, linked to multiple neoplasms | https://www.uniprot.org/uniprotkb/P01127 |
| *PDGFD* | Q9GZP0 | Potentially coronary artery disease, colorectal carcinoma | https://www.uniprot.org/uniprotkb/Q9GZP0 |
| *PENK* | P01210 | Dyskinesia, potentially colorectal cancer | https://www.uniprot.org/uniprotkb/P01210 |
| *PLAU* | P00749 | Alzheimer's disease, Quebec platelet disorder, cardiovascular diseases. Prognosis in B-cell lymphoma | https://www.uniprot.org/uniprotkb/P00749 |
| *PLAUR* | Q03405 | Neoplasms, neoplasm invasiveness and metastasis. Potentially MAGI | https://www.uniprot.org/uniprotkb/Q03405 |
| *POMC* | P01189 | Obesity, proopiomelanocortin deficiency, mental disorders, Cushing’s syndrome and EAS | https://www.uniprot.org/uniprotkb/P01189 |
| *PROS1* | P07225 | Thrombophilia, protein S deficiency. Risk of venous thromboembolism | https://www.uniprot.org/uniprotkb/P07225 |
| *PTGDS* | P41222 | Kidney function and CVD | https://www.uniprot.org/uniprotkb/P41222 |
| *PTGS2* | P35354 | Endometriosis, colonic neoplasms, polymorphisms in rectal cancer, multiple neoplastic processes | https://www.uniprot.org/uniprotkb/P35354 |

| | | | |
|---|---|---|---|
| *PTHLH* | P12272 | Humoral hypercalcemia of malignancy, peripheral chondrosarcoma, malignant neoplasm of breast, prostate | https://www.uniprot.org/uniprotkb/P12272 |
| *PTN* | P21246 | Tumor vascularization | https://www.uniprot.org/uniprotkb/P21246 |
| *RBP3* | P10745 | Retinitis pigmentosa, mutation in retinal dystrophy | https://www.uniprot.org/uniprotkb/P10745 |
| *RELN* | P78509 | Neuropsychiatric disorders | https://www.uniprot.org/uniprotkb/P78509 |
| *RGMA* | Q96B86 | Monitor treatment of neuro-degenerative disease | https://www.uniprot.org/uniprotkb/Q96B86 |
| *RLN1* | P04808 | NSCLC classification | https://www.uniprot.org/uniprotkb/P04808 |
| *RLN2* | P04090 | Prostatic neoplasms. NSCLC classification | https://www.uniprot.org/uniprotkb/P04090 |
| *SERPINA1* | P01009 | Early emphysema, alpha 1-antitrypsin deficiency, lung diseases | https://www.uniprot.org/uniprotkb/P01009 |
| *SERPINB2* | P05120 | Malignant neoplasm of breast, stomach. Favorable cancer prognosis | https://www.uniprot.org/uniprotkb/P05120 |
| *SERPINE1* | P05121 | Plasminogen activator Inhibitor-1 deficiency, female urogenital diseases and pregnancy complications. Subtyping of breast cancer | https://www.uniprot.org/uniprotkb/P05121 |
| *SLPI* | P03973 | Renal function | https://www.uniprot.org/uniprotkb/P03973 |
| *SOD3* | P08294 | Cardiovascular diseases, polymorphisms in COPD | https://www.uniprot.org/uniprotkb/P08294 |
| *SPARC* | P09486 | Osteogenesis Imperfecta. Potentially pancreatic cancer prognosis | https://www.uniprot.org/uniprotkb/P09486 |
| *SPARCL1* | Q14515 | Colorectal cancer | https://www.uniprot.org/uniprotkb/Q14515 |
| *SULF2* | Q8IWU5 | Malignant neoplasm of breast. Potentially lung cancer | https://www.uniprot.org/uniprotkb/Q8IWU5 |
| *TF* | P02787 | Congenital atransferrinemia, prostate cancer | https://www.uniprot.org/uniprotkb/P02787 |
| *TFF1* | P04155 | Stomach carcinoma. Breast cancer prognostic marker | https://www.uniprot.org/uniprotkb/P04155 |
| *TFF2* | Q03403 | Marker for aiding diagnosis of lobular endocervical glandular hyperplasia and gastric-type adenocarcinoma | https://www.uniprot.org/uniprotkb/Q03403 |
| *TFF3* | Q07654 | Thyroid follicular carcinoma | https://www.uniprot.org/uniprotkb/Q07654 |
| *TGFB1* | P01137 | Camurati-Engelmann disease, fibrosis, asthma. Congenital, cardiovascular and respiratory tract diseases. | https://www.uniprot.org/uniprotkb/P01137 |
| *TGFB2* | P61812 | Loeys-Dietz Syndrome, aortic aneurysm and aortic dissection. Osteoporosis | https://www.uniprot.org/uniprotkb/P61812 |
| *TGFBI* | Q15582 | Some cancers, such as liver cancer. Corneal dystrophy | https://www.uniprot.org/uniprotkb/Q15582 |
| *TIMP1* | P01033 | Cardiovascular and respiratory tract diseases. | https://www.uniprot.org/uniprotkb/P01033 |
| *TIMP3* | P35625 | Age related macular degeneration, Fundus dystrophy. Gastric adenocarcinoma | https://www.uniprot.org/uniprotkb/P35625 |
| *TNC* | P24821 | Inflammation, deathless, asthma. | https://www.uniprot.org/uniprotkb/P24821 |
| *TNFAIP6* | P98066 | Mild osteoarthritis | https://www.uniprot.org/uniprotkb/P98066 |
| *TNXB* | P22105 | Potentially rheumatoid arthritis | https://www.uniprot.org/uniprotkb/P22105 |
| *TRH* | P20396 | Hypothyroidism and hyperthyroidism, endocrine system diseases | https://www.uniprot.org/uniprotkb/P20396 |
| *VEGFA* | P15692 | Diabetic conditions, retinal vein occlusion, malignant tumor of colon, psoriasis | https://www.uniprot.org/uniprotkb/P15692 |
| *WNT5A* | P41221 | Robinow syndrome, inflammatory diseases, asthma, malignant neoplasm of lung | https://www.uniprot.org/uniprotkb/P41221 |
| *WNT6* | Q9Y6F9 | Osteoporosis diagnosis and therapy | https://www.uniprot.org/uniprotkb/Q9Y6F9 |
| *WNT9A* | O14904 | Idiopathic carpal tunnel syndrome. May suppress colorectal cancer cells | https://www.uniprot.org/uniprotkb/O14904 |
